# Diverse p120RasGAP interactions with doubly phosphorylated partners EphB4, p190RhoGAP, and Dok1

**DOI:** 10.1016/j.jbc.2023.105098

**Published:** 2023-07-27

**Authors:** Kimberly J. Vish, Amy L. Stiegler, Titus J. Boggon

**Affiliations:** 1 Department of Molecular Biophysics and Biochemistry, Yale University, New Haven, Connecticut, USA; 2 Department of Pharmacology, Yale University, New Haven, Connecticut, USA; 3 Department of Yale Cancer Center, Yale University, New Haven, Connecticut, USA

**Keywords:** p120RasGAP, *RASA1*, ephrin receptor, phosphotyrosine, Src homology 2

## Abstract

RasGAP (p120RasGAP), the founding member of the GTPase-activating protein (GAP) family, is one of only nine human proteins to contain two SH2 domains and is essential for proper vascular development. Despite its importance, its interactions with key binding partners remains unclear. In this study we provide a detailed viewpoint of RasGAP recruitment to various binding partners and assess their impact on RasGAP activity. We reveal the RasGAP SH2 domains generate distinct binding interactions with three well-known doubly phosphorylated binding partners: p190RhoGAP, Dok1, and EphB4. Affinity measurements demonstrate a 100-fold weakened affinity for RasGAP-EphB4 binding compared to RasGAP-p190RhoGAP or RasGAP-Dok1 binding, possibly driven by single *versus* dual SH2 domain engagement with a dominant N-terminal SH2 interaction. Small-angle X-ray scattering reveals conformational differences between RasGAP-EphB4 binding and RasGAP-p190RhoGAP binding. Importantly, these interactions do not impact catalytic activity, implying RasGAP utilizes its SH2 domains to achieve diverse spatial-temporal regulation of Ras signaling in a previously unrecognized fashion.

Control of small GTPase enzymatic activity by GTPase-activating proteins (GAPs) was first revealed with the identification of the archetypal Ras-GAP protein RasGAP (p120RasGAP, GAP 1; encoded by *RASA1*) (1, 2). RasGAP was found to contribute a conserved arginine guanidino group to the active site of Ras, facilitating significant acceleration in the rate of gamma-phosphate cleavage from GTP and consequent cycling of Ras from the GTP-bound active state to the GDP-bound inactive state (3, 4, 5). “Arginine finger”–based catalysis was subsequently found to be highly conserved across GAP proteins for Ras, Rho, and other small GTPases (6, 7), and GAP proteins for Ras were found to use this well-conserved fold to bind and correctly coordinate Ras-bound GTP with their arginine finger residue (8). However, in contrast to the highly conserved GAP domain, each Ras GAP protein family contains a unique array of modular domains associated with functions such as correct spatial-temporal localization or enzymatic regulation (6). This is exemplified by RasGAP which possesses domains whose influence on signaling remain unresolved, including two N-terminal Src homology 2 (SH2) domains and Src homology 3 (SH3), Pleckstrin homology, and PKC conserved 2 (C2) domains (Fig. 1*A*) (9).

**Figure 1:**
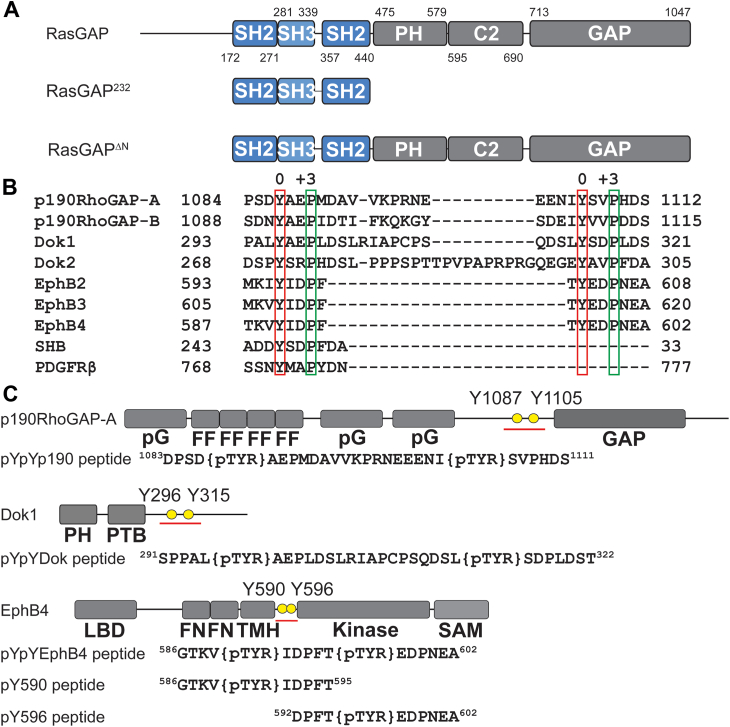
**RasGAP and its phosphorylated binding partners.***A*, domain map of RasGAP and protein constructs used in this study. RasGAP (UniProt ID: P20936) domains are indicated by the following: SH2, Src homology 2; SH3, Src homology 3; PH, Pleckstrin homology; C2, PKC conserved domain 2; and GAP, GTPase-activating protein. Start and end residues of each domain are indicated. RasGAP^232^ contains SH2, SH3, and SH2 domains, and RasGAP^ΔN^ contains all folded domains. *B*, alignment of phosphotyrosine (position 0) and +3 prolines of known p120RasGAP binding partners. The alignment was made using MAFFT (86). Phosphotyrosine highlighted in *red* and Proline at +3 highlighted in *green*. Residue numbers indicated. *C*, domain maps of p190RhoGAP-A, Dok1, and EphB4. p190RhoGAP-A (UniProt ID: Q9NRY4) domains indicated by pG for pseudoGTPase and FF named for two conserved phenylalanine residues in domain. Dok1 (UniProt ID: Q99704) phosphotyrosine-binding domain (PTB) indicated by PTB. EphB4 (UniProt ID: P54760) domains indicated by the following: LBD, ligand-binding domain; FN, fibronectin; TMH, transmembrane helix; SAM, sterile alpha motif; and kinase. Sequences and locations of phosphopeptides used in this study indicated.

RasGAP is an essential protein that is required for developmental, neonatal, and pathological angiogenesis, and lymphatic, lymphovenous and venous valve development (10, 11, 12, 13, 14). This is thought to be due to signaling downstream of the EphB4 receptor tyrosine kinase, which controls correct patterning and differentiation of vascular endothelial cells (15, 16, 17). In endothelial cells, EphB4 can downregulate rather than upregulate Ras signaling, achieved by direct recruitment of RasGAP to the activated receptor (18, 19). Consequently, mutation or loss of *EPHB4* or *RASA1* genes are demonstrated to dysregulate vascular development in mice (11) and zebrafish (12), as well as other mammalian organisms (10, 20). RasGAP and EphB4 are clinically important in vascular disorders where mutations in *RASA1* and *EPHB4* are causal for the most common neonatal neurovascular disorder, vein-of-Galen malformations, and one of the major neurovascular disorders, capillary malformation-arteriovenous malformation syndrome (21, 22, 23, 24). Importantly, the mutated RasGAP and EphB4 proteins are disrupted in their ability to suppress Ras activity, with consequential impacts on vascular structures and circuitry in mutant animals (21, 24, 25, 26). The similar phenotypes of mutant RasGAP and EphB4 may point to the potential importance of their interaction, though this has not been fully established.

Direct interaction between RasGAP and EphB4 has been observed *in vitro*, and mutational studies have revealed that the basis for RasGAP recruitment to EphB receptors is by RasGAP SH2–mediated recognition of phosphotyrosine residues within the juxtamembrane region of the activated EphB receptor (19, 27, 28). These juxtamembrane phosphorylation sites are known regulators of Eph receptor kinase activity and their mutation prevents the receptor activation (29, 30, 31). Surprisingly, however, the specific details of the RasGAP–EphB interaction remain unknown, and importantly, the stoichiometry of binding between the doubly phosphorylated EphB juxtamembrane region and the tandem SH2 domains of RasGAP has not been assessed. A tandem phosphotyrosine–SH2 complex would likely result in a significantly tighter and long-lived interaction than a single phosphotyrosine–SH2–mediated complex, with implications for the longevity of impacts on EphB and Ras signaling.

RasGAP is also recruited *via* its SH2 domains to the p190RhoGAP family and to the Dok family of adaptor proteins (32, 33, 34, 35, 36). RasGAP recruitment to p190RhoGAP is important for stress-fiber formation and cell motility (37, 38, 39) and is often regarded as an example of Rho-Ras crosstalk (28, 38, 39, 40, 41, 42, 43), while the Dok family of proteins are recruited downstream of receptor tyrosine kinases and modulate Ras signaling through RasGAP (34, 44, 45). Both p190RhoGAP and Dok1 become phosphorylated downstream of receptor tyrosine kinases (28, 36, 41). The two tyrosine residues in p190RhoGAP’s middle domain are known to be phosphorylated by Src and Arg (35, 46) and these phosphorylation sites have been observed extensively by mass spectrometry analyses (47, 48). Dok1’s phosphorylation landscape is more complicated since there are multiple tyrosines in Dok1’s extended C-terminal tail that get phosphorylated downstream of receptor tyrosine kinase activation (28, 36, 45). While many of these have been shown to interact with RasGAP, Y296 and Y315 are of particular interest due to their high sequence similarity to p190RhoGAP. A bisphosphorylated peptide of these sites is able to compete with full-length Dok1 to bind RasGAP (49). Similar to the RasGAP–EphB receptors interaction, the nature of the stoichiometry of RasGAP–p190RhoGAP or RasGAP–Dok interactions had not been addressed until recent work showed that doubly phosphorylated p190RhoGAP maintains an extremely tight interaction with the tandem SH2 domains of RasGAP (50). This contrasts with single SH2–phosphotyrosine interactions of the individual RasGAP SH2 domains and singly phosphorylated p190RhoGAP peptides, which are approximately 15- to 30-fold weaker (51, 52). These studies suggest that RasGAP interaction with its phosphotyrosine binding partners could represent a decision point for spatial-temporal control of Ras pathways, with tandem binding of RasGAP’s dual SH2 domains to dual phosphotyrosine residues providing a more sustained interaction than single SH2-phosphotyrosine recognition. Thus, single-*versus* dual- SH2 domain recognition of tyrosine phosphorylated partners could allow tuning of Ras regulation and Ras signaling pathways.

Disentangling the mechanisms of recruitment and interaction of signaling partners downstream of receptor tyrosine kinases remains a challenge, but for RasGAP, the dual SH2 domains represent a potential mechanism for tuning signaling. We therefore investigated the interactions of RasGAP with three of its doubly phosphorylated binding partners: EphB4, p190RhoGAP-A, and Dok1. Although these are all tandem phosphotyrosine binding partners, we observe a wide range of affinities, with RasGAP binding to p190RhoGAP-A or Dok1 approximately 100-fold tighter than it binds EphB4. Our data indicate that this difference in affinity may be driven by single *versus* dual SH2–phosphotyrosine interactions, with p190RhoGAP-A and Dok1 coordinating both SH2 domains of RasGAP, but EphB4 binding primarily a single SH2, and with lower affinity. Tight binding of dual phosphotyrosine partners induces conformational changes in the SH2-SH3-SH2 region, but interestingly we find that neither high- nor low-affinity interactions alter GAP activity. Our study therefore provides new insights into the mechanisms by which RasGAP distinguishes its binding partners and indicates that the dual SH2 domains of RasGAP function to selectivity regulate its recruitment to binding partners.

## Results

Nine of the 111 SH2-containing genes in the human genome contain two SH2 domains (53). These dual SH2-containing proteins include protein tyrosine phosphatases SHP1 and SHP2, nonreceptor tyrosine kinases ZAP-70 and Syk, the signaling effector phospholipase C-γ1, the alpha regulatory subunit of PI3K, and p120RasGAP. As shown for many of these proteins, and exemplified for SHP2 (54), the presence of conserved dual SH2 domains can play important roles in signaling and regulation. Therefore, we wondered if RasGAP’s dual SH2 domains may play a similarly important role in signaling of this important Ras regulator. Sequence analysis of RasGAP proteins over evolution demonstrates the presence of two conserved SH2 domains as far back as Trichoplax (Fig. S1). This evolutionary conservation of SH2 domains seems to suggest functional importance for the tandem arrangement, however not all of RasGAP’s phosphotyrosine–mediated binding partners contain multiple phosphotyrosines. For example, some binding partners are thought to bind *via* a single phosphorylation site, such as platelet-derived growth factor (55, 56, 57) and SH2 domain-containing adapter protein B (58). In contrast, other partners, including the EphB, p190RhoGAP, and Dok families, have the potential to interact *via* multiple phosphorylation sites simultaneously (28, 35, 49). Interactions of the dual SH2 domains of RasGAP may therefore provide an intricate platform for RasGAP to distinguish its partner interactions. If this is indeed the case, the primary sequence of the phosphorylated binding partners may provide insights into the selectivity of partner recognition. We therefore conducted sequence alignment of the phosphotyrosine motifs of known RasGAP binding partners.

We aligned the nine best-studied RasGAP binding partners for which interactions were previously validated by either point mutation or domain deletion (12, 27, 28, 35, 49, 51, 52, 55, 58) (Fig. 1*B*), and we found that all of these validated RasGAP-binding partner phosphotyrosine motifs display a proline residue at the +3 position, where phosphotyrosine is located at position 0. This result correlates with the previously identified selectivity preference of the RasGAP SH2 domains, which find a strong preference for proline at the +3 position (59, 60). Among these, we note that three families have two -pY-x-x-P- phosphotyrosine sites in close proximity, but with different spacing. These partners, the EphB receptor family, the p190RhoGAP family, and the Dok family, display phosphotyrosines separated by six amino acids (EphB receptor), 18 amino acids (p190RhoGAP), and 19 to 28 amino acids (Dok) (Fig. 1*B*). The differences in separation of the phosphotyrosine residues suggest that there may be variability in the binding mode of these doubly phosphorylated binding partners. We therefore decided to assess the affinity of interactions between RasGAP and synthesized peptide examples from these doubly phosphorylated partner protein families.

**Figure 2:**
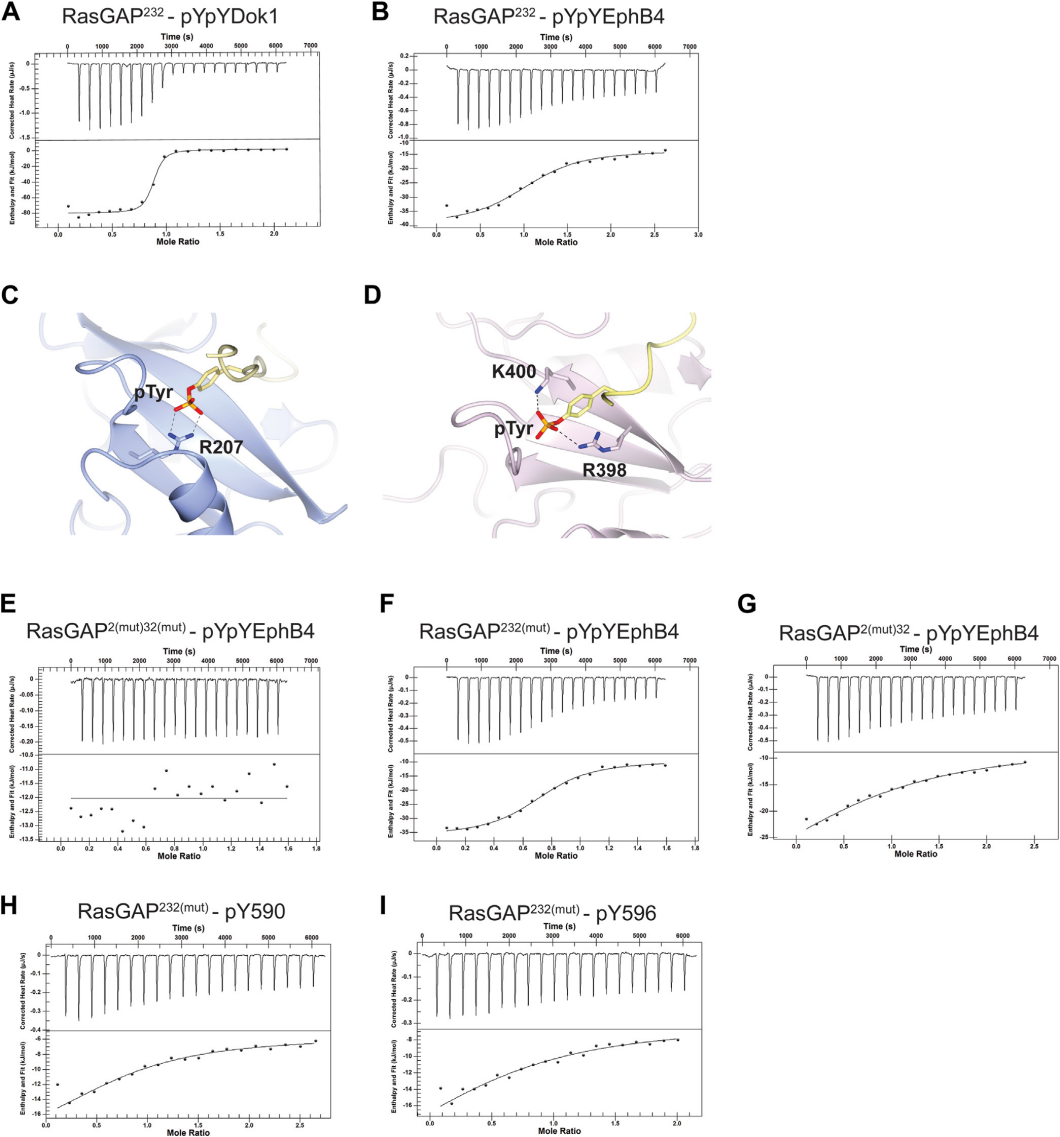
**Binding isotherms for RasGAP interactions with phosphorylated partners.** Example thermograms and binding isotherms for WT RasGAP interactions with (*A*) doubly phosphorylated Dok1 and (*B*) doubly phosphorylated EphB4 phosphopeptides. *C*, cartoon diagram of the phosphotyrosine-binding site of RasGAP N-SH2 illustrating residue R207. PDB ID: 6PXC (51). *D*, cartoon diagram of the phosphotyrosine-binding site of RasGAP C-SH2 illustrating residues R398 and K400. PDB ID: 6WAY (52). *E*–*G*, example thermograms and binding isotherms for mutant RasGAP interactions with pYpYEphB4 phosphopeptide. *H* and *I*, example thermograms and binding isotherms for RasGAP^232(mut)^ with singly phosphorylated EphB4 phosphopeptides.

We expressed and purified the region of RasGAP encompassing both SH2 domains with a construct that includes the N-SH2, SH3, and C-SH2 domains (residues 174–444, termed RasGAP^232^ (50)) (Figs. 1*A* and S2*A*). Separately, we synthesized peptides containing both phosphotyrosine residues for EphB4 and Dok1 (Fig. 1*C*). Our previous isothermal titration calorimetry (ITC) study assessed the affinity of RasGAP^232^ for doubly phosphorylated p190RhoGAP-A peptide and found a K_d_ of 10 ± 6 nM for tandem engagement of both phosphotyrosines by both SH2 domains (50). In our current study, titration of the doubly phosphorylated Dok1 peptide against RasGAP^232^ reveals a similar K_d_ of 30 ± 20 nM (Fig. 2*A*, Table 1, Fig. S3, Table S1), revealing p190RhoGAP-A and Dok1 to display similarly tight double pY binding. In contrast, titration of the doubly phosphorylated EphB4 peptide against RasGAP^232^ reveals a K_d_ of 2 ± 0.7 μM (Fig. 2*B*, Table 1, Fig. S3, Table S1). This is a striking difference of approximately 100-fold and suggests that RasGAP^232^ engages the EphB4 peptide in a different manner to the p190RhoGAP-A and Dok1 peptides (Tables 1 and S1, Fig. S3).

**Table 1:** Isothermal titration calorimetry measurements for p120RasGAP with phosphotyrosine-containing binding partners

Sample cell	Syringe	K_d_ (μM)	n	ΔH (kJ/mol)	ΔS (J/mol∗K)	Citation
Doubly phosphorylated peptide titrations						
RasGAP^232^	pYpY EphB4	2 ± 0.7	1 ± 0.1	−24 ± 3	30 ± 10	This work
RasGAP^2(mut)32(mut)^	pYpY EphB4	N.D.				This work
RasGAP^2(mut)32^	pYpY EphB4	40 ± 20	0.9 ± 0.2	−33 ± 6	−30 ± 20	This work
RasGAP^232(mut)^	pYpY EphB4	1 ± 0.3	0.75 ± 0.02	−25 ± 1	28 ± 5	This work
RasGAP^232^	pYpY Dok1	0.03 ± 0.02	0.8 ± 0.1	−78 ± 3	−120 ± 14	This work
RasGAP^232^	pYpY p190RhoGAP-A	0.01 ± 0.006	0.6 ± 0.1	−130 ± 20	−281 ± 77	(50)
Singly Phosphorylated Peptide Titrations						
RasGAP^232(mut)^	pY590 EphB4	16 ± 7	0.8 ± 0.1	−15.5	40 ± 18	This work
RasGAP^232(mut)^	pY596 EphB4	21 ± 3	0.6 ± 0.2	−22 ± 4	15 ± 13	This work
RasGAP^N-SH2^	pY1105 p190RhoGAP-A	0.3 ± 0.1	0.68 ± 0.01	−70 ± 10	−95 ± 40	(51)
RasGAP^C-SH2^	pY1087 p190RhoGAP-A	0.15 ± 0.04	0.91 ± 0.08	−60 ± 7	−70 ± 30	(52)

Thermodynamic data for phosphotyrosine peptide titrations against purified WT or mutant RasGAP^232^ proteins or the individual SH2 domains. Doubly phosphorylated peptides indicated as pYpY. Singly phosphorylated peptides for p190RhoGAP-A and EphB4 indicated with the phosphorylated residue number. N.D. means not determined (there was no significant binding). Data were fit to the independent binding model in NanoAnalyze (TA Instruments).

One possibility for the difference in binding affinity of EphB4 compared to the other dually phosphorylated peptides is that only a single SH2 domain engages EphB4 at any time. This is supported by the observation that the K_d_ of binding, 2 μM, is similar to other reported individual SH2 phosphotyrosine interactions (for examples see (60)) and the evidence of only one inflection point in the doubly phosphorylated EphB4–binding isotherm (Figs. 2*B* and S3). To assess this hypothesis, we generated two RasGAP^232^ mutants that disrupt the phosphotyrosine-binding sites of either the N- or C-SH2 domains and conducted further ITC experiments. For the N-terminal SH2, we mutated the canonical “FLVR” arginine, R207 to alanine, termed RasGAP^2(mut)32^ (Fig. 2*C*), which has previously been shown to be sufficient to abolish phosphotyrosine binding by this domain (51). For the ‘FLVR-unique” C-terminal SH2, we introduced a double mutation R398E/K400E, to abrogate phosphotyrosine binding guided by our previous work (52), termed RasGAP^232(mut)^ (Fig. 2*D*). We additionally generated a double mutant of both SH2 domains, RasGAP^2(mut)32(mut)^, and purified each of these proteins (Fig. S2, *B*–*D*). We found that titration of the double mutant RasGAP^2(mut)32(mut)^ with doubly phosphorylated EphB4 peptide revealed no detectable binding, illustrating that binding requires functional SH2 domains and rules out a measurable phosphotyrosine-independent component (Fig. 2*E*, Table 1, Fig. S3, Table S1). We then assessed phosphopeptide binding of the single mutant SH2 domains. Titration of the C-SH2 mutant RasGAP^232(mut)^ with doubly phosphorylated EphB4 peptide revealed a K_d_ of 1 ± 0.3 μM (Fig. 2*F*, Table 1, Fig. S3, Table S1). This is similar to the K_d_ of 2 ± 0.7 μM observed for titration of this peptide into WT RasGAP^232^. In contrast, titration of the N-SH2 mutant RasGAP^2(mut)32^ with doubly phosphorylated EphB4 peptide demonstrated a substantially weaker K_d_ of 40 ± 20 μM (Fig. 2*G*, Table 1, Fig. S3, Table S1). Together, these data suggest that the interaction with doubly phosphorylated EphB4 is predominantly driven by the N-terminal SH2 domain of RasGAP (Tables 1 and S1).

To attempt to parse the specificity of RasGAP for the more N- or C-terminal EphB juxtamembrane phosphotyrosines pY590 and pY596, we assessed the affinity of RasGAP^232(mut)^ for shorter synthesized EphB4 peptides corresponding to either of the two phosphotyrosines (Fig. 1*C*). We find that both of the singly phosphorylated peptides bind about 10-fold weaker to RasGAP^232(mut)^ than the doubly phosphorylated EphB4 peptide, and we do not observe large differences between them (K_d_ of 16 ± 7 μM for the pY590 peptide and 21 ± 3 μM for the pY596 peptide) (Table 1, Fig. 2, *H* and *I*, Table S1, Fig. S3). We interpret these data to indicate that the N-terminal SH2 domain is primarily responsible for direct RasGAP binding to Ephrin-B receptors, and that the presence of the two phosphotyrosines in close proximity allows for an increase in affinity to RasGAP through an avidity effect by allowing N-SH2 to bind to either pY site (to “slide” between pY sites). Alternatively, the C-terminal SH2 may contribute to a second weaker interaction in a tandem interaction. However, a second inflection point in the isotherm is not observed, suggesting the interaction is primarily N-SH2 driven. These analyses suggest that even though these receptors are doubly phosphorylated, the EphB receptors bind to RasGAP with approximately 100-fold weaker affinity than doubly phosphorylated p190RhoGAP or Dok protein binding to RasGAP, which places them in a different binding class (low affinity). This low-affinity interaction represents a primarily single N-SH2-dominant interaction.

**Figure 3:**
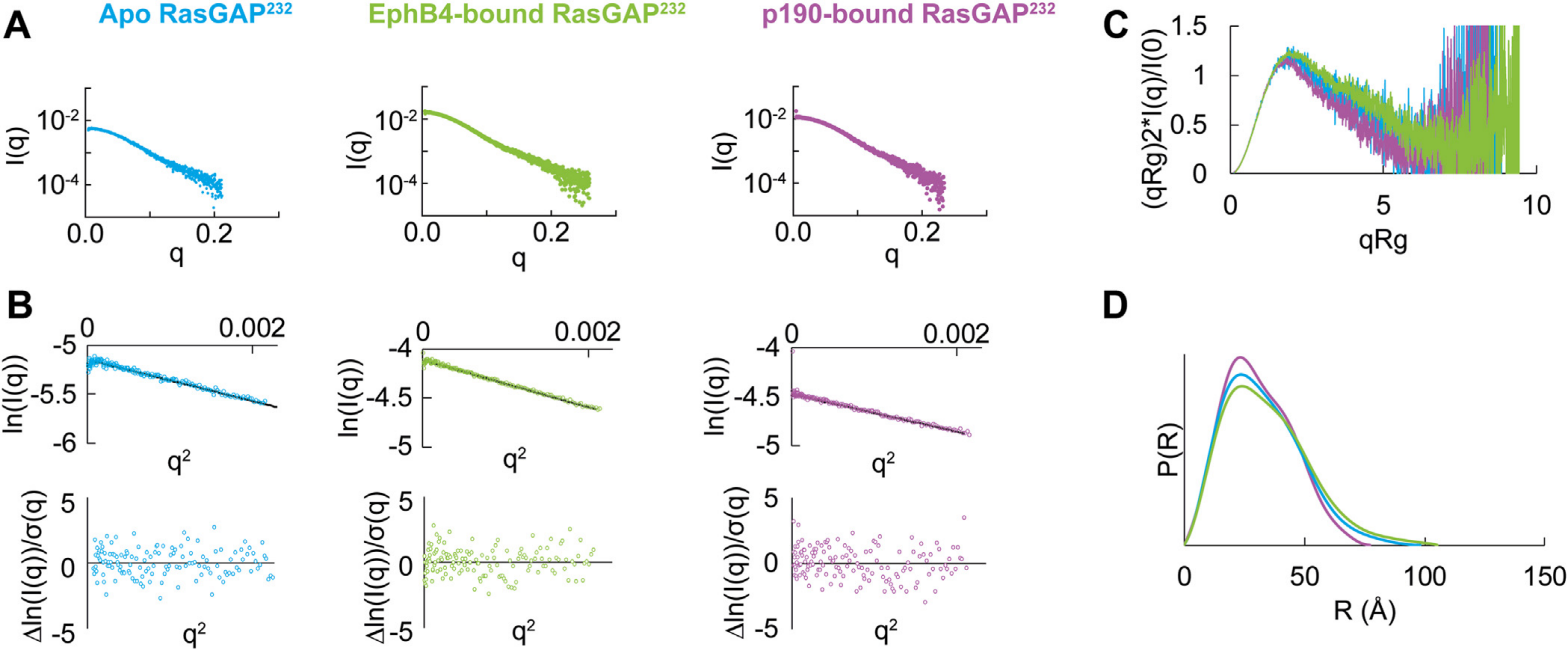
**Small-angle scattering of RasGAP SH2-SH3-SH2 region when bound to doubly phosphorylated partners.***A*, scattering profiles of apo and peptide-bound RasGAP^232^. *B*, Guinier analysis of the scattering profiles with residuals plotted. Guinier plots show no deviations from linearity. *C*, Kratky analysis of EphB4-bound RasGAP^232^ was compared to apo and p190-bound RasGAP^232^. EphB4-bound RasGAP^232^ is more similar to apo RasGAP^232^ than p190-bound RasGAP^232^. *D*, pair distribution functions of all three samples. The EphB4-bound sample has a longer tail closely resembling the apo RasGAP^232^. In all panels *blue* indicates apo RasGAP^232^, *green* indicates EphB4-bound RasGAP^232^, and *purple* indicates p190-bound RasGAP^232^.

Phosphotyrosine motif binding to RasGAP has long been considered to be a potential mechanism to achieve targeted alterations in Ras signaling (41, 49, 61, 62) and recent studies revealed that doubly phosphorylated p190RhoGAP-A induces changes in RasGAP^232^ conformation (50). We therefore wondered if the variability of binding affinity to RasGAP for EphB, p190RhoGAP, and Dok family members suggests differences in RasGAP conformational responses to phosphopeptide binding. To test this hypothesis, we conducted small-angle X-ray scattering (SAXS) to probe the in-solution effect of binding. We prepared apo and peptide-bound samples at concentrations approximately 100 times above K_d_ to ensure stable complex formation, and after elution, we confirmed the presence of peptide by native gel shifts. Previous work showed that when RasGAP^232^ engages a doubly phosphorylated p190RhoGAP-A peptide the sample compacts compared to apo RasGAP^232^ (50). Our data confirm this effect, with Kratky and pair distribution analyses both indicating significant compaction of RasGAP^232^ when bound to doubly phosphorylated p190RhoGAP-A peptide accompanied by an approximately 21 Å change in D_max_ between the two samples (Fig. 3, Table 2, Fig. S4, Table S2). In contrast, when we assess the scattering of RasGAP^232^ bound to doubly phosphorylated EphB4 we find no major conformational changes. Kratky analysis of RasGAP^232^ bound to EphB4 indicates flexibility similar to the apo sample (Fig. 3*C*) and likewise in *R*_g_ and D_max_ (Tables 2 and S2). We additionally observe the pair distribution function for both apo and EphB4-bound RasGAP^232^ to be comparable shapes (Fig. 3*D*). These data reveal that engagement of RasGAP^232^ with EphB4 does not significantly impact the local conformation of RasGAP^232^. Overall, the engagement of RasGAP^232^ with its phosphorylated binding partners therefore seems to result in a variety of local conformations dependent on the phosphorylated binding partner.

**Table 2:** Small-angle X-ray scattering analyses of RasGAP and its interactions with doubly phosphorylated binding partner peptides

Protein construct	R_g_ (Å)	D_max_ (Å)	Volume of correlation (Vc) MW (kDa)	MALS MW (kDa)	Theoretical MW (kDa)
RasGAP^232^					
Apo RasGAP^232^	25.1 ± 0.1	98	31.1	33.0 ± 0.5	31.4
p190-bound RasGAP^232^	23.9 ± 0.06	77	31.0	35.0 ± 0.2	34.9
EphB4-bound RasGAP^232^	26.8 ± 0.1	105	32.1	35.2 ± 0.6	33.6
RasGAP^ΔN^					
Apo RasGAP^ΔN^	39.5 ± 0.1	140	100.3	106.9 ± 0.5	103.4
p190-bound RasGAP^ΔN^	40.1 ± 0.1	151	108.9	108.7 ± 0.6	106.9
Dok1-bound RasGAP^ΔN^	40.5 ± 0.1	146	107.5	109.8 ± 0.5	107
EphB4-bound RasGAP^ΔN^	39.2 ± 0.1	137	116.6	106.8 ± 0.4	105.6

**Figure 4:**
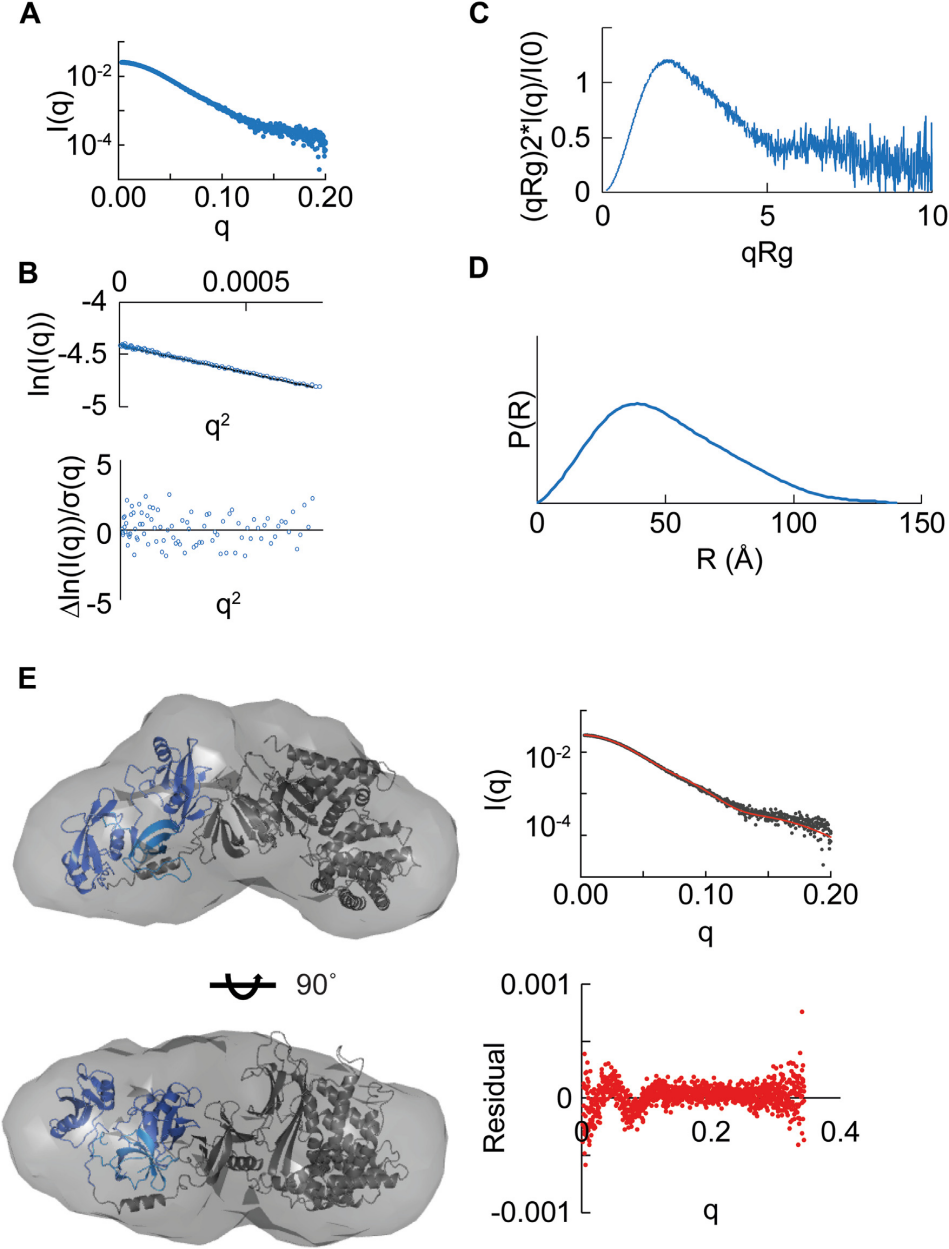
**Small-angle X-ray scattering reveals the overall shape of RasGAP.***A*, scattering profile of apo RasGAP^ΔN^. Profile generated from averaging scattering profiles over the sample peak. *B*, Guinier linearization. There are no deviations from linearity. *C*, Kratky analysis. While flexibility is present, overall, the sample is compact. *D*, pair distribution function reveals a globular shape with a tail that represents flexibility. *E*, overlay of electron density from apo RasGAP^ΔN^ scattering using DENSS (81) with the AlphaFold (63) model of human p120RasGAP (UniProt ID: P20936; AF-P20936-F1-model_v2.pdb) using residues 165 to 1047 (63). Domains are colored according to Figure 1*A*. On *right-hand-side*, FoXS (87) analysis of the theoretical scattering of AlphaFold’s prediction of RasGAP’s residues 165 to 1047 (*red line*) to the experimental apo RasGAP^ΔN^ scattering (*gray dots*). Residuals are plotted. χ^2^ = 1.15. DENSS, DENsity from Solution Scattering.

**Figure 5:**
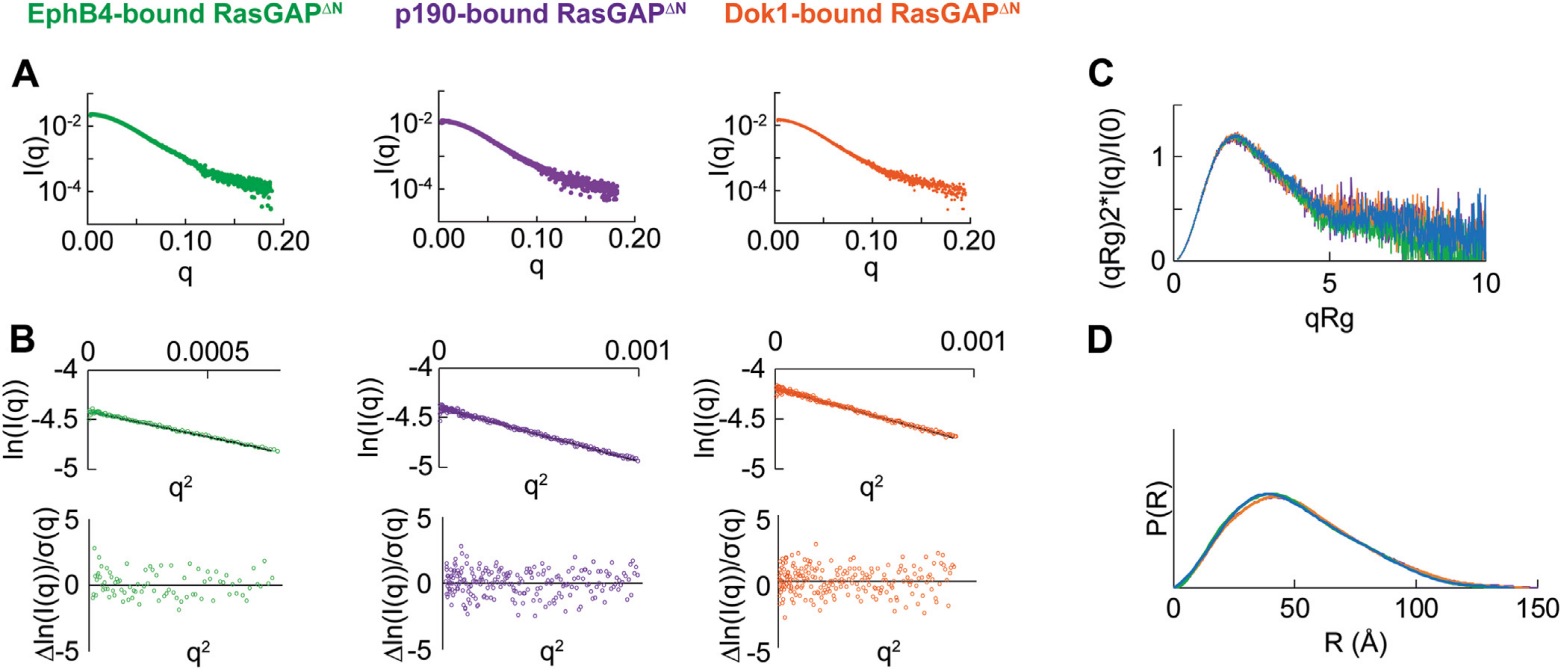
**Small-angle X-ray scattering for peptide-bound RasGAP**^**ΔN**^**.***A*, profiles of peptide-bound RasGAP^ΔN^ samples. *B*, Guinier plots of each sample to determine sample quality with plotted residuals. All samples lack deviations from linearity which reflects no protein aggregation. *C*, Kratky plots of all samples indicates similar flexibility. *D*, pair distribution function showing all samples display similar overall shape. In all panels *blue* indicates apo RasGAP^ΔN^, *green* indicates EphB4-bound RasGAP^ΔN^, *purple* indicates p190-bound RasGAP^ΔN^, and *orange* indicates Dok1-bound RasGAP^ΔN^.

R_g_ was calculated by Guinier analysis (Figs. 3*B*, 4*B*, and 5*B*). D_max_ was derived from pair distribution function analysis (Figs. 3*D*, 4*D*, and 5*D*).

We next asked whether differences in binding mode might translate to overall conformational changes in RasGAP. To achieve this, we expressed and purified near full-length RasGAP lacking its flexible N-terminal region (residues 174–1047, termed RasGAP^ΔN^, Figs. 1*A* and S2, *E* and *F*). SAXS for apo RasGAP^ΔN^ reveals a semicompact structure with a D_max_ of 140 Å, and Kratky analysis indicates more rigidity than would be expected for a multidomain protein if it were in an extended conformationally flexible conformation (Fig. 4, *A*–*D*, Tables 2 and S2, Fig. S5). This agrees with our electron density reconstruction of RasGAP^ΔN^, which mostly reveals a globular shape. Interestingly, comparing this envelope to the structure of RasGAP (174–1047) predicted by AlphaFold (63) results in similar shapes (χ^2^ = 1.15, where lower values indicate better fit) (Fig. 4*E*). Correlation of SAXS with the AlphaFold model for RasGAP is highly suggestive of interdomain interactions within RasGAP, which may provide constraints to the protein’s global conformation. The SAXS analysis of apo RasGAP^ΔN^ therefore reveals a compact and conformationally stable overall structure with interdomain interactions even in the absence of peptide-binding partner.

To assess whether partner engagement results in large-scale conformational rearrangement of RasGAP, we bound RasGAP^ΔN^ to doubly phosphorylated peptides of EphB4, p190RhoGAP-A, and of Dok1 by preparing samples that are approximately 10-fold above the K_d_ and conducted SAXS studies for these complexes. We find that *R*_g_, D_max_, Kratky plots, and pair distribution function for each of the samples suggests both globally similar conformations and flexibility when compared to the apo sample (Fig. 5, Table 2, Fig. S5). Conformational changes that occur in the SH2-SH3-SH2 region of RasGAP do not seem to translate to global conformational movements observable by SAXS, suggesting these movements are masked by the flexibility contributed by the Pleckstrin homology, C2, and GAP domains of the near full-length protein. Therefore, a higher resolution technique will be necessary to tease out peptide-induced conformational movements in the RasGAP^ΔN^ construct.

**Figure 6:**
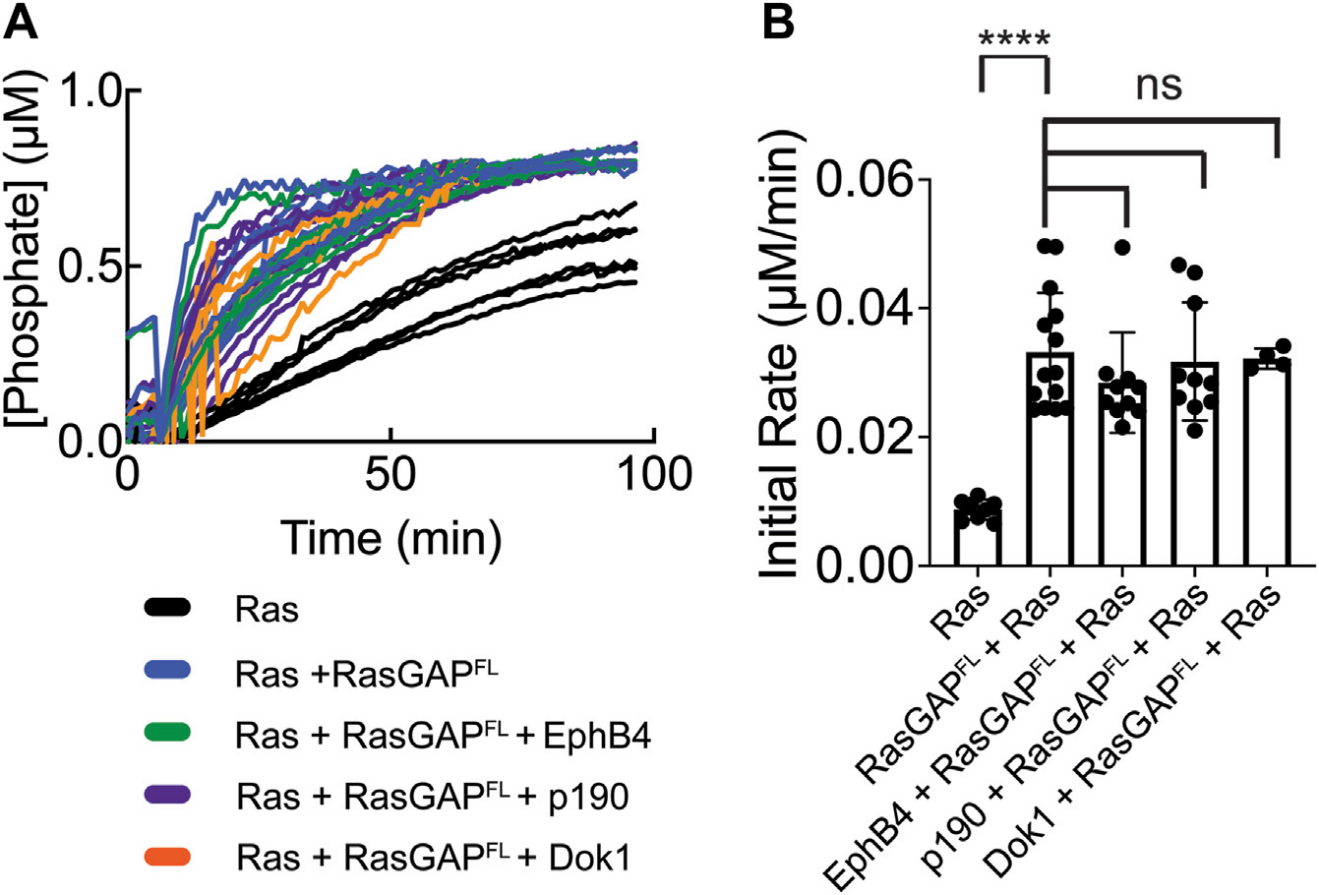
***In vitro* GAP assays.***A*, GAP assay for full-length RasGAP activation of GTP-loaded H-Ras. As indicated, reactions included GTP-H-Ras (GTPase domain), 25 nM RasGAP^FL^, and 50 μM EphB4, 50 μM 50 μM p190RhoGAP-A, or 50 μM Dok1 doubly phosphorylated peptide. Reactions started at 7 min by addition of Mg^2+^. *B*, peptides do not impact GAP activity. Initial rates calculated for each reaction using only the first 10 min after addition of Mg^2+^, where the phosphate release rate was linear. For statistics, an unpaired nonparametric Mann-Whitney test was used (GraphPad Prism). ∗∗∗∗ indicates *p* = <0.0001. “ns” indicates not significant (*p* > 0.05). n = 4 to 14 for each sample with each replicate rate plotted, bars represent average, error bars represent SD. GAP, GTPase-activating protein.

Finally, to probe whether the conformational movements in the SH2-SH3-SH2 region nonetheless impact catalytic activity *in vitro*, we conducted GAP assays. Previous studies have suggested that changes in RasGAP activity occur on engagement with binding partners (41, 49), and early studies proposed the SH2 domains to directly modulate GAP activity (61, 62). Our SAXS data for RasGAP^232^ may provide a basis for how this is achieved: by inducing local changes in 232 conformations upon peptide engagement. Therefore, we purified full-length RasGAP (residues 1–1047, termed RasGAP^FL^, Fig. S2*G*) and assessed activity against purified H-Ras GTPase domain preloaded with GTP (residues 1–167, Fig. S2*H*) in a single turnover fluorescence-based phosphate sensor assay that monitors phosphate release in real time (64). We find that RasGAP^FL^ activates H-Ras, as expected (Fig. 6) (61, 62). We then incubated RasGAP^FL^ with each of the doubly phosphorylated peptides corresponding to EphB4, p190RhoGAP-A, and Dok1 at concentrations high enough to drive complex formation (50 μM) and conducted GAP assays against H-Ras. When compared to RasGAP^FL^ neither the low-affinity EphB4 peptide nor the high-affinity p190RhoGAP-A and Dok1 peptides significantly alter *in vitro* GAP activity of RasGAP toward H-Ras. We conclude that the engagement of phosphotyrosine peptides primarily guides selectivity toward binding partners rather than intramolecular regulation of GAP function.

## Discussion

Targeted control of Ras signaling pathways are essential for normal cellular function. This control is achieved by an array of mechanisms, with the GAP playing major roles in controlling normal function. Many of the early studies on GAP function were conducted on the foundational member of the family, RasGAP, and indeed it was studies of RasGAP that revealed the atomic-level structure of the Ras-targeting GAP fold and arginine finger catalysis mechanism, along with similar studies on RhoGAP with the Rho GTPase (4, 6, 7, 65). Nonetheless, the domain architecture of RasGAP has remained less well understood, and importantly the significance of its dual SH2 domains obscure. In this study, we reveal the role of the tandem SH2 domains to be a filter for selective binding to its doubly phosphorylated partner proteins. We show interactions with some doubly phosphorylated binding partners, but not others, can induce local conformational changes in RasGAP, and that recruitment to doubly phosphorylated binding partners does not directly impact *in vitro* GAP activity. We also reveal the first conformational understanding of the overall structure of RasGAP. These studies establish that the dual SH2 domains of RasGAP help regulate a more intricate functional readout than has previously been appreciated.

RasGAP is the only GAP protein for Ras family members that contains SH2 domains, providing it a unique ability to directly bind phosphotyrosine partners downstream of receptor tyrosine kinases (6). RasGAP is similarly unusual among the 111 human SH2 domain–containing proteins as it is one of only nine to contain dual SH2 domains (53), and its engagement of phosphotyrosine by its C-terminal SH2 domain is different from all other SH2 domains (which we termed “FLVR-unique”) (52, 66). These unusual features are conserved in RasGAP from humans to placozoa (Fig. S1) (52), suggesting functional importance of the SH2 domains, and indeed, many interaction partners have been identified, some of which are thought to bind *via* a single phosphotyrosine site (*e.g.*, platelet-derived growth factor and SH2 domain-containing adapter protein B (55, 56, 57, 58)), and others which contain tandem tyrosine phosphorylation sites (*e.g.*, the Ephrin-B receptors, the p190RhoGAP family, and the Dok adaptor proteins (28, 32, 49)). The SH2 domains are therefore important conserved functional contributors to RasGAP signaling.

The two RasGAP SH2 domains have both been demonstrated to display a binding preference for -pY-x-x-P- motifs (28, 51, 52, 59, 60). Sequence analysis of EphB, the p190RhoGAP, and the Dok proteins demonstrates that each has two closely spaced -Y-x-x-P- motifs which become phosphorylated, supporting the understanding that these three protein groups tandemly engage the SH2 domains of RasGAP (28, 35, 49). Our closer inspection of these sites (Fig. 1), however, reveals that whereas the p190RhoGAP and Dok phosphotyrosines are 18 or more residues distal from one another, the EphB phosphotyrosines are separated by only six residues. We postulated, based on our previous crystal structure (50), that the difference in phosphotyrosine spacing may impact RasGAP binding and provide an unrecognized molecular-level mechanism to tune partner affinities, conformational reorganization, or GAP activity. In short, the SH2 domains of RasGAP may provide a gating mechanism to control signaling in a previously unrecognized manner.

We assessed these potential impacts of binding and reveal that doubly phosphorylated regions of Dok1 and p190RhoGAP-A (50) display nanomolar affinities for RasGAP and induce local conformational changes in the SH2-SH3-SH2 region. The tight affinities for SH2-mediated interactions are unusual and indicate dual coordination of phosphosites by the dual SH2 domains, with affinities approximately 15- to 30-fold tighter than individual SH2–phosphotyrosine interactions (60) (Table 1). In contrast, the doubly phosphorylated region of EphB4 displays affinities for RasGAP similar to single SH2 domain–phosphotyrosine interactions (60). Indeed, our ITC studies support the hypothesis that this interaction is mediated primarily by the N-terminal SH2 domain. However, the Eph receptor low-affinity binding class, like the p190RhoGAP/Dok high-affinity binding class, does seem to utilize both phosphotyrosines to increase binding affinity, but unlike the tandem engagement of the p190RhoGAP/Dok binding class, an avidity effect seems to drive increased affinity for RasGAP by the tandemly phosphorylated Eph receptor class. We find that individual interactions with either of the EphB4 phosphotyrosine residues are possible, and that binding affinity of the doubly phosphorylated peptide is enhanced 8- to 10-fold, (a more modest effect than the 15–30 fold observed for the p190RhoGAP group). These differences in affinity support our hypothesis that spacing between phosphorylated tyrosine residues controls the affinity. We therefore conclude that multiple classes of RasGAP binding partners exist, partially differentiated by phosphotyrosine spacing.

SH2 domains are commonly considered to be protein modules (67) and linear depictions of protein domain architecture (*e.g.*, in Fig. 1*A*) can often reinforce the notion of domains functioning independently of one another, which is more likely to occur when proteins fold in a “beads-on-a-string” arrangement. In contrast, coordinated responses to protein–protein interactions are more likely to occur in the context of a compact arrangement. Our SAXS study provides the first near full-length assessment of RasGAP’s structure and demonstrates that it does not maintain a “beads-on-a-string” organization of its six domains, but rather a compact, globular architecture. Our experimental evidence correlates well with the current AlphaFold model for RasGAP (63), and both biophysical and *in silico* data support RasGAP as a largely globular protein with interdomain interactions.

The global conformation of RasGAP raises possibilities for concerted conformational responses to protein–protein interactions, and in the context of the SH2-SH3-SH2 region, we find that the high-affinity interactions with p190RhoGAP-A and Dok1 induce local changes similar to functionally important conformational rearrangements of tandem SH2 domains in other proteins (68, 69). We do not observe large-scale conformational changes on high-affinity peptide binding in near full-length RasGAP, but this may potentially be explained by the resolution limits of SAXS. In contrast, the low-affinity interaction with EphB4 does not significantly alter the conformation of the SH2-SH3-SH2 region, and likewise we observe no changes in near full-length RasGAP upon EphB4 binding. Global conformational changes therefore potentially provided a rationale for previously observed alterations in GAP activity upon Dok1 or p190RhoGAP binding (41, 49), but in our *in vitro* analysis of purified full-length RasGAP, we did not observe alterations in *in vitro* GAP activity on peptide addition. As the previous studies were conducted in cell lysates (41, 49) we hypothesize that additional factors may be important to achieve similar alterations in activity. Our conformational studies therefore provide a framework for us to reconcile these data. We hypothesize that induced conformational changes in RasGAP may occur without globally altering its shape or enzymatic activity, but that well-spaced tandemly phosphorylated partners may potentially stabilize cryptic-binding site(s) with to-be-determined impacts on signaling occurring uniquely for tandemly bound but not singly bound RasGAP.

Our study of the EphB4–RasGAP interaction also reveals new insights into the Ephrin-B group. The EphBs are unusual receptor tyrosine kinases because Ras signaling is downregulated on their activation in endothelial cells (18, 19). The mechanism for this is thought to be mediated, at least in part, by RasGAP recruitment to the phosphorylated juxtamembrane region linking to Ras inactivation (19, 28, 70). We show that RasGAP–EphB4 interaction is likely driven *via* the N-terminal SH2-phosphotyrosine recruitment and not the equal N-terminal SH2/C-terminal SH2 recruitment previously assumed (12, 19, 22, 28). This may have implications for understanding EphB receptor regulation, as affinity and dissociation rate of a juxtamembrane-bound SH2 domain would necessarily impact dephosphorylation and inactivation steps. Furthermore, single SH2 binding to EphB receptors also raises the possibility that the second SH2 domain may be involved in recruitment to a third protein, allowing RasGAP to act as a molecular tether to the EphB receptors.

RasGAP binding partners are multidomain proteins (Fig. 1*A*) and therefore there remains the possibility that contributions from regions outside of the phosphotyrosine motifs may contribute to affinity, conformational, and even enzymatic regulation of RasGAP function. Previous work from cell lysate Dok1 and p190 suggested downregulation of RasGAP activity toward Ras (41, 49), but we did not observe similar findings with purified samples. Furthermore, p190 binding to RasGAP is hypothesized to expose a surface on RasGAP’s SH3 domain that is occluded in RasGAP’s apo state (35), an effect which may not be observable by SAXS. There is, nonetheless, precedence for noncanonical SH2 domain secondary interactions. The selectivity of phospholipase C-γ1′s N-terminal SH2 domain for activated fibroblast growth factor receptor relies on an extended secondary interaction surface (71). Further studies should assess the potential for secondary sites outside of the canonical SH2-mediated phosphotyrosine interactions of RasGAP and its partners and whether the diversity of domain architecture of the binding partners hides a conserved cryptic secondary site.

The functional importance of the SH2–phosphotyrosine interactions of RasGAP and its partners has been demonstrated extensively. Phenylalanine mutations prevent RasGAP binding in cell lysates to p190 (35) and removal of the phosphotyrosines of Dok1 prevent pulldown with RasGAP in cell lysates (49). In the Ephrin B receptors, although the juxtamembrane tyrosine residues regulate EphB activation (29, 31), their mutation to glutamate reduces, but not eliminate RasGAP recruitment (19), but this may result from Dok1 or Nck acting as a bridge between RasGAP and EphB4 (28). Our study therefore provides further evidence of the critical nature of the phosphotyrosine interactions for the binding partner interactions of RasGAP.

**Figure 7:**
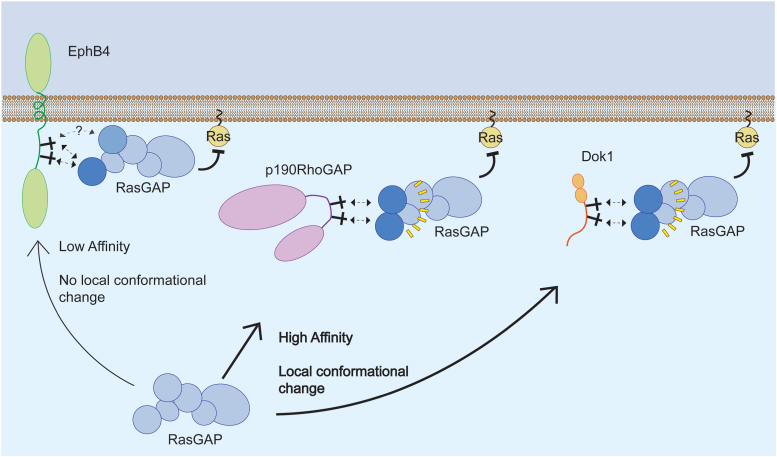
**The tandem SH2 domains of RasGAP act as a signaling selectivity filter.** Low-affinity interactions of RasGAP with EphB receptors are driven by a single phosphotyrosine interaction with the N-terminal RasGAP SH2 domain. High-affinity interactions of RasGAP with p190RhoGAP and Dok proteins are driven by tandem phosphotyrosine interactions with both SH2 domains, inducing a local conformational change in RasGAP.

RasGAP recruitment to binding partners has long been considered important for its spatial-temporal regulation of Ras signaling (19, 72), but the nature of the interactions and the roles of the unusual dual SH2 domains have remained elusive. Our work suggests the dual SH2 domains may play more complex roles than simple SH2-phosphotyrosine–binding sites, and that these domains may fine-tune signaling by acting as wide dynamic range selectivity filters for binding partners to facilitate complex regulation of signaling. This may have implications in downstream MAP kinase signaling, as a tighter affinity may allow RasGAP to be recruited to a Ras signaling hub for a longer period of time than a weaker affinity might. There also could be implications in Ras-Rho crosstalk, for which RasGAP is known to be important (32, 38, 51, 73), since engaging only one RasGAP SH2 domain leaves the other available for other binding partners. Together, this study therefore provides new insights into binding and conformation of RasGAP and lead us to construct a schematic understanding of how phosphotyrosine engagement with doubly phosphorylated partners may occur and the SH2-mediated interactions providing a filter for both binding partner affinity and stabilization (Fig. 7). Delineating the molecular and functional basis for protein interactions remains a critical aspect of understanding how signal transduction pathways are controlled and our findings both provide key insights into the importance of the SH2 domains of RasGAP and suggest that the functional roles of the adaptor domains of RasGAP may be significantly underappreciated.

## Experimental procedures

### Expression constructs and protein purification

RasGAP^ΔN^, RasGAP^232^ (50), and H-Ras^1-167^ were cloned into a modified pET bacterial expression vector using BamHI and XhoI restriction sites. Site-directed mutagenesis was performed using QuikChange protocol (Agilent). All mutants were purified using the same protocol as their WT counterparts. RasGAP^232^ and RasGAP^ΔN4CS^ contain four cystine to serine mutations (C261, C236, C372, and C402) to remove disulfide-linked multimerization, which caused protein heterogeneity as described in (50). All plasmid constructs were transformed into Rosetta (DE3) cells, which were grown shaking at 37 °C until *A*_600_ 0.6 to 0.8, when protein expression was induced with 0.1 to 0.25 mM IPTG. Cells were incubated overnight at 18 °C shaking and harvested by centrifugation at 2000*g* for 30 min, and pellets resuspended in lysis buffer (50 mM Hepes pH 8, 500 mM NaCl). Cells were then lysed *via* the addition of 25 μg/ml lysozyme, three freeze-thaw cycles, and sonication, then spun down at 5000*g* for 1 h. The supernatant was added to Ni-NTA agarose beads (Thermo Fisher Scientific) and rocked at 4 °C for 1 h.

### RasGAP^232^ purification

Bead-bound His-tagged RasGAP^232^ was eluted in a stepwise gradient of 20 mM, 40 mM, 100 mM, 250 mM, and 500 mM imidazole in lysis buffer. Fractions containing RasGAP^232^ were pooled and mixed with hexahistidine-tagged Tobacco Etch Virus (TEV), and dialyzed overnight against lysis buffer to proteolyze the hexahistidine tag and remove imidazole. The solution was then added back to nickel beads and rocked for 1 h at 4 °C to remove TEV and uncleaved protein. The flowthrough containing tagless RasGAP^232^ was concentrated and buffer exchanged into 20 mM Tris pH 8 to reduce NaCl concentration to 50 mM. Anion-exchange chromatography was then performed using either MonoQ 5/50 GL (Cytiva) or ResourceQ (1 ml, Cytiva) columns and buffers A and B which were 20 mM Tris pH 8 and 20 mM Tris pH 8, 1 M NaCl, respectively. Next, size-exclusion chromatography (SEC) was performed using HiLoad 16/600 Superdex 75 prep grade (Cytiva) with 20 mM Tris pH 8, 250 mM NaCl buffer.

### H-Ras^1-167^ purification

Bead-bound His-tagged H-Ras^1-167^ was eluted in a stepwise gradient of 20 mM, 40 mM, 100 mM, 250 mM, and 500 mM imidazole in lysis buffer. Fractions containing H-Ras^1-167^ were pooled and mixed with hexahistidine-tagged TEV and dialyzed overnight against lysis buffer to proteolyze the hexahistidine tag and remove imidazole. The solution was then added back to nickel beads and rocked for 1 h at 4 °C to remove TEV and uncleaved protein. The flowthrough containing tagless H-Ras^1-167^ was concentrated and buffer exchanged into 20 mM Tris pH 8 to reduce NaCl concentration to 50 mM. Anion-exchange chromatography was then performed using either MonoQ 5/50 GL (Cytiva) or ResourceQ (1 ml, Cytiva) columns and buffers A and B which were 20 mM Tris pH 8 and 20 mM Tris pH 8, 1 M NaCl, respectively. Next, SEC was performed using HiLoad 16/600 Superdex 75 prep grade (Cytiva) with 20 mM Tris pH 8, 150 mM NaCl buffer.

### RasGAP^ΔN^ purification

Bead-bound His-tagged RasGAP^ΔN^ was eluted in a stepwise gradient of 10 mM, 20 mM, 100 mM, 250 mM, and 500 mM imidazole in lysis buffer. Fractions containing RasGAP^ΔN^ were pooled and dialyzed overnight in buffer containing 20 mM Tris pH 8, 150 mM NaCl to decrease the salt concentration. The next day the RasGAP^ΔN^ sample was concentrated and buffer exchanged into 20 mM Tris pH 8 to reduce NaCl concentration further to 50 mM. Anion-exchange chromatography was then performed using either MonoQ 5/50 GL (Cytiva) or ResourceQ (1 ml, Cytiva) columns and buffers A and B which were 20 mM Tris pH 8.5 and 20 mM Tris pH 8.5, 1 M NaCl, respectively. Next, SEC was performed using HiLoad 16/600 Superdex 200 prep grade (Cytiva) with 20 mM Tris pH 8, 150 mM NaCl buffer.

### Peptide synthesis

Synthetic peptides were purchased from GenScript and contain N-terminal acetylation and C-terminal amidation for stability. The doubly phosphorylated EphB4 peptide (UniProt ID P54760) used in ITC and SAXS experiments contains residues 586 to 602 (GTKV[pTyr]IDPFT[pTyr]EDPNEA). The singly phosphorylated EphB4 peptides pY590 and pY596 used in ITC experiments contain the residues 586 to 595 (GTKV[pTyr]IDPFT) and residues 592 to 602 (DPFT[pTyr]EDPNE), respectively. The p190RhoGAP peptide (UniProt ID Q9NRY4) used in SAXS experiments contains the residues 1083 to 1111 (SD[pTyr]AEPMDAVVKPRNEEENI[pTyr]SVPH). The Dok1 peptide (UniProt ID Q99704) used in ITC and SAXS experiments contains the residues 291 to 322 (SPPAL[pTyr]AEPLDSLRIAPCPSQDSL[pTyr]SDPLDST). Lyophilized peptides were reconstituted in water, and less soluble peptides (pYpYEphB4 and pY590) were spiked with 20 mM Tris pH 8 or 10 mM NaOH to increase solubility. Concentrations of stock peptides are as follows: pYpY190 stock concentration is 10 mM; pYpYEphB4 stock concentration is 2.5 mM; Dok1 stock concentration is 5.5 mM; pY596 stock concentration is 10 mM; and pY590 stock concentration is 8 mM.

### Isothermal titration calorimetry

Purified RasGAP^232^ protein and solubilized peptides were dialyzed overnight against buffer containing 20 mM Tris pH 8, 250 mM NaCl in Slide-A-Lyzer 3 ml 10 kDa molecular weight cut off cassettes (Spectra-Por) and Micro Float-A-Lyzer 0.1 to 0.2 ml, 0.1 to 0.5 Da molecular weight cut off devices (Spectra-Por), respectively. Protein and peptide concentrations were determined by *A*_280_ measured on a Nanodrop Lite (Thermo Fisher Scientific) instrument using extinction coefficients of 45,840 M^−1^ cm^−1^ for RasGAP^232^ (and mutants), 917 M^−1^ cm^−1^ for the doubly phosphorylated EphB4 peptide, and 458.5 M^−1^ cm^−1^ for the singly phosphorylated EphB4 peptides as determined by (74). Dok1 peptide concentration was confirmed using Amino Acid Analysis performed at UC Davis Molecular Structure Facility. Samples were centrifuged for 10 min at 4 °C and degassed for 7 min using a Degassing Station (TA Instruments). ITC experiments were performed on a Nano ITC (TA Instruments) by loading protein into the 190 μl sample cell and titrating 20 times with 2.5 μl peptide in the titration syringe. Concentrations for sample cell and syringe for each trial are included in Table S1. The data were analyzed in NanoAnalyze software (TA Instrument) using blank (constant) and independent models (https://www.tainstruments.com/itcrun-dscrun-nanoanalyze-software/).

### Small angle X-ray scattering

After purification, 0.5 mM peptide was added to 0.1 mM RasGAP^232^, and samples were buffer exchanged into SAXS buffer (20 mM Tris pH 8, 350 mM NaCl, 1 mM DTT) using a Superdex 75 10/300 increase GL (Cytiva). For RasGAP^ΔN^ samples, 0.12 mM peptide was added to 0.04 mM RasGAP^ΔN^ and buffer exchanged into SAXS buffer (20 mM Tris pH 8, 150 mM NaCl, 1 mM DTT) using a Superdex 200 10/300 increase GL (Cytiva). Apo and EphB4-bound RasGAP^ΔN^ scattering data were collected on the WT RasGAP^ΔN^ protein, while the p190RhoGAP-A-bound and Dok1-bound samples used the RasGAP^ΔN4CS^ protein (Fig. S1*F*) to remove nonspecific disulfide effects.

SAXS was performed at BioCAT (beamline 18-ID at the Advanced Photon Source, Argonne National Laboratories) with in-line SEC to separate sample from aggregates and other contaminants thus ensuring optimal sample quality, and multiangle light scattering (MALS), dynamic light scattering (DLS), and refractive index measurement for additional biophysical characterization (SEC-MALS-SAXS). The samples (see Table S2 for concentrations and volumes) were loaded on a Superdex 200 Increase 10/300 Gl column (Cytiva) run by a 1260 Infinity II HPLC (Agilent Technologies) at 0.6 ml/min. The flow passed through (in order) the Agilent UV detector, a MALS detector and a DLS detector (DAWN Helios II, Wyatt Technologies), and a refractive index detector (Optilab T-rEX, Wyatt), followed by the SAXS flow cell. The flow cell consisted of a 1 mm ID quartz capillary with ∼20 μm walls. A coflowing buffer sheath was used to separate sample from the capillary walls, helping prevent radiation damage (75). Scattering intensity was recorded using an EIGER2 XE 9M (Dectris) detector which was placed 3.6 m from the sample giving us access to a q-range of 0.003 Å^−1^ to 0.42 Å^−1^. Exposures of 0.5 s were acquired every 1 s during elution, and data was reduced using BioXTAS RAW 2.1.1 (76). Buffer blanks were created by averaging regions flanking the elution peak and subtracted from exposures selected from the elution peak to create the I(q) *versus* q curves used for subsequent analyses. Molecular weights and hydrodynamic radii were calculated from the MALS and DLS data, respectively using the ASTRA 7 software (Wyatt) (https://www.wyatt.com/products/software/astra.html#astra-5). Radiation damage was monitored using CORMAP (77) implemented in BioXTAS RAW. Data processing was performed for Guinier fit and molecular weight estimations using BioXTAS RAW, and pair distribution function using GNOM (78). RAW uses MoW and Vc M.W. methods (79, 80). Electron density reconstructions were performed in RAW v2.1.3, which implements the program DENsity from Solution Scattering (81).

### Enzymatic assays

H-Ras was preloaded with GTP by mixing purified H-Ras^1-167^ protein, 10 mM EDTA, and 10-fold excess GTP in 20 mM Tris pH 8, 100 mM NaCl for 1 h at room temperature. Excess GTP was removed by SEC using a Superdex 75 10/300 Gl column and exchange buffer (20 mM Tris pH 8, 100 mM NaCl, 10 mM EDTA). Loading efficiency was checked by heating 0.1 nmol of H-Ras at 95 °C for 15 min to denature protein, spinning down to remove protein, and loading the supernatant containing nucleotide onto a MonoQ 5/50 GL (Cytiva) column. Once on the column, the associated nucleotide was eluted using NaCl and the conductivity was compared to known GTP and GDP standards, adapted from (82, 83, 84). After, 0.8 μM GTP-H-Ras was mixed with 25 nM full-length RasGAP in the absence or presence of 50 μM peptide to ensure full binding and 1 μM phosphate sensor (commercially purchased from Thermo Fisher Scientific, adapted by (64, 85)) in kinetic buffer (20 mM Tris pH 8, 50 mM NaCl, 0.01% Triton X-100, 1 mM tris(2-carboxyethyl)phosphine). Reaction volume was 20 μl, performed in 384-well black microplate. Fluorescence excitation and emission settings were 430 nm and 450 nm, respectively with a bandwidth of 10 nm, and readings were taken in 1-min intervals. After measuring six times to collect a baseline, the reaction was started by adding 5 mM MgCl_2_. The readings were taken on a Synergy H1 plate reader (BioTek Agilent) using Gen5 3.11 software (https://www.agilent.com/en/product/microplate-instrumentation/microplate-instrumentation-control-analysis-software/imager-reader-control-analysis-software/biotek-gen5-software-for-detection-1623227). Data were then normalized to fluorescence as suggested by (64) and initial rates determined by measuring the slope during the first 10 min of assay time. Peptide alone did not affect Ras GTP hydrolysis in absence of GAP. *p* values calculated using an unpaired nonparametric Mann-Whitney test (GraphPad Prism v. 9.4.0) (https://www.graphpad.com/).

## Data availability

All collected SAXS profiles and pair distribution functions are uploaded to the SASBDB under the accession codes SASDRJ6, SASDRK6, SASDRL6, SASDRE6, SASDRF6, SASDRG6, and SASDRH6.

## Supporting information

This article contains supporting information (88).

## Conflict of interest

The authors declare that they have no conflicts of interest with the contents of this article.
