# Supplementary material for: Diverse p120RasGAP interactions with doubly phosphorylated partners EphB4, p190RhoGAP, and Dok1
Source: J Biol Chem. 2023 Jul 27;299(9):105098. doi: 10.1016/j.jbc.2023.105098 (PMC10470053; doi:10.1016/j.jbc.2023.105098)
Supplement: Supporting Table S1 and S2 and Figures S1–S5 [file mmc1.pdf]

Supplemental Material for:

Diverse RasGAP interactions with doubly phosphorylated partners, EphB4, p190RhoGAP and Dok1

Kimberly J. Vish[1], Amy L. Stiegler[2], and Titus J. Boggon[1,2,3*]

From: Departments of [1]Molecular Biophysics and Biochemistry and [2]Pharmacology, and the [3]Yale Cancer Center, Yale University, New Haven, CT, USA

* To whom correspondence should be addressed: titus.boggon@yale.edu

Short Title: RasGAP interactions with EphB4, p190RhoGAP and Dok1

Table S1. Isothermal titration calorimetry measurements of each trial. RasGAP$^{2(mut)32(mut)}$ titrated with pYpYEphB4 did not produce meaningful data indicating very weak or no binding.

Trial	Sample	Syringe	blank	K_d	n	ΔH	K_a	-TΔS	ΔG	ΔS
Sample cell: **RasGAP^{232}** Syringe: **pYpY EphB4**										
1	0.02	0.22	-8.6	1.3	1.0	-25.1	0.8	-8.6	-33.7	28.9
2	0.027	0.22	-8.1	2.4	1.0	-25.5	0.4	-6.6	-32.1	22.2
3	0.026	0.22	-7.4	2.7	1.1	-26.1	0.5	-5.7	-31.8	19.1
4	0.03	0.15	-6.2	1.5	0.9	-19.4	0.7	-14.0	-33.3	46.8
**average**			**-7.6**	**2.0**	**1.0**	**-24.0**	**0.6**	**-9**	**-33**	**30**
**stdev**			**1.0**	**0.7**	**0.1**	**3**	**0.2**	**4**	**1**	**10**
Sample cell: **RasGAP$^{2(mut)32}$** Syringe: **pYpY EphB4**										
1	0.032	0.24	-5.6	25	0.7	-35.3	0.04	8.0	-26.3	-26.7
2	0.032	0.24	-4.6	32	1.0	-32.8	0.03	7.1	-25.6	-24.0
3	0.03	0.25	-4.1	60	0.9	-39.2	0.02	15.2	-24.1	-50.8
4	0.03	0.25	-4.1	38	1.2	-25.1	0.03	-0.1	-25.2	0.5
**average**			**-5**	**40**	**0.9**	**-33**	**0.03**	**8**	**-25**	**-30**
**stdev**			**0.7**	**20**	**0.2**	**6**	**0.01**	**6**	**1**	**20**
Sample cell: **RasGAP$^{232(mut)}$** Syringe: **pYpY EphB4**										
1	0.03	0.15	-3.6	1.1	0.8	-26.6	0.9	-7.4	-34.0	24.9
2	0.03	0.15	-3.5	1.5	0.7	-26.8	0.7	-6.5	-33.3	21.8
3	0.03	0.15	-3.7	1.0	0.8	-24.7	1.0	-9.4	-34.2	31.7
4	0.03	0.225	-3.7	0.8	0.7	-24.7	1.0	-10.2	-34.8	34.0
**average**			**-4**	**1.1**	**0.8**	**-26**	**1.0**	**-8**	**-34**	**28**
**stdev**			**0.1**	**0.3**	**0.02**	**1**	**0.2**	**1**	**0.60**	**6**
Sample cell: **RasGAP$^{232(mut)}$** Syringe: **pY596 EphB4**										
1	0.03	0.25	-3.0	24	0.7	-19.5	0.04	-6.9	-26.4	23.1
2	0.03	0.189	-2.9	22	0.8	-20.1	0.04	-6.6	-26.6	22.0
3	0.0293	0.189	-3.0	18	0.4	-26.9	0.05	-0.2	-27.1	0.8
**average**			**-3.0**	**21**	**0.6**	**-22**	**0.05**	**-5**	**-26.7**	**15**
**stdev**			**0.1**	**3**	**0.2**	**4**	**0.008**	**4**	**0.4**	**13**
Sample cell: **RasGAP$^{232(mut)}$** Syringe: **pY590 EphB4**										
1	0.03	0.25	-3	24.2	0.7	-19.5	0.04	-6.9	-26.4	23.1
2	0.03	0.25	-3.5	14.6	0.8	-15.8	0.07	-11.8	-27.6	40.0
3	0.03	0.25	-4	9.7	0.8	-11.2	10	-17.4	-28.6	58.3
**average**			**-3.5**	**16**	**0.8**	**-16**	**0.07**	**-12**	**-28**	**40**
**stdev**			**0.5**	**7**	**0.07**	**4**	**0.03**	**5**	**1**	**18**
Sample cell: **RasGAP^{232}** Syringe: **pYpY Dok1**										
1	0.02	0.133	-1.1	0.016	0.9	-75	63	30	-44	-102
2	0.02	0.133	0.3	0.047	0.8	-81	21	39	-42	-131
3	0.02	0.133	-0.2	0.023	0.7	-78	43	35	-44	-116
**average**				**0.03**	**0.8**	**-78**	**40**	**35**	**-43**	**-120**
**stdev**				**0.02**	**0.1**	**3**	**20**	**4**	**1**	**14**

Table S2. SAXS statistics. Statistics for small-angle X-ray scattering data collection, processing and analysis of RasGAP and its interactions with doubly phosphorylated binding partner peptides. $RasGAP^{\Delta N4CS}$ contains cysteine to serine mutations to remove unwanted disulfied effects[50].

	Apo $RasGAP^{232}$	EphB4-bound $RasGAP^{232}$	p190-bound $RasGAP^{232}$	Apo $RasGAP^{\Delta N}$	EphB4-bound $RasGAP^{\Delta N}$	p190-bound $RasGAP^{\Delta N4CS}$	Dok1-bound $RasGAP^{\Delta N4CS}$
**Sample Details**							
Organism		human		human	human	human	human
Source		*E. coli*		*E. coli*	*E. coli*	*E. coli*	*E. coli*
Sequence of protein construct	MHHHHHHGSLVPRSENLYFQGSTAPPTNQWYHGKLDRTIAEERLRQAGKSGSYLIRESDRRPGSFVLSFLSQMNVVNHFRIIAMSGDYYIGGRRFSSLSDLIGYYSHVSSLLKGEKLLYPVAPPEPVEDRRRVRAILPYTKVPDTDEISFLKGDMFIVHNELEDGWMWVTNLRTDEQGLIVEDLVEEVGREEDPHEGKIWFHGKISKQEAYNLLMTVGQVSSFLVRPSDNTPGDYSLYFRTNENIQRFKISPTPNNQFMMGGRYYNSIGDIIDHYRKEQIVEGYYLKEPVPMQ			MHHHHHHGSLVPRSENLYFQGSTAPPTNQWYHGKLDRTIAEERLRQAGKSGSYLIRESDRRPGSFVLSFLSQMNVVNHFRIIAMCGDYYIGGRRFSSLSDLIGYYSHVSCLLKGEKLLYPVAPPEPVEDRRRVRAILPYTKVPDTDEISFLKGDMFIVHNELEDGWMWVTNLRTDEQGLIVEDLVEEVGREEDPHEGKIWFHGKISKQEAYNLLMTVGQVCSFLVRPSDNTPGDYSLYFRTNENIQRFKICPTPNNQFMMGGRYYNSIGDIIDHYRKEQIVEGYYLKEPVPMQDQEQVLNDTVDGKEIYNTIRRKTKDAFYKNIVKKGYLLKKGKGKRWKNLYFILEGSDAQLIYFESEKRATKPKGLIDLSVCSVYVVHDSLFGRPNCFQIVVQHFSEEHYIFYFAGETPEQAEDWMKGLQAFCNLRKSSPGTSNKRLRQVSSLVLHIEEAHKLPVKHFTNPYCNIYLNSVQVAKTHAREGQNPVWSEEFVFDDLPPDINRFEITLSNKTKKSKDPDILFMRCQLSRLQKGHATDEWFLLSSHIPLKGIEPGSLRVRARYSMEKIMPEEEYSEFKELILQKELHVVYALSHVCGQDRTLLASILLRIFLHEKLESLLLCTLNDREISMEDEATTLFRATTLASTLMEQYMKATATQFVHHALKDSILKIMESKQSCELSPSKLEKNEDVNTNLTHLLNILSELVEKIFMASEILPPTLRYIYGCLQKSVQHKWPTNTTMRTRVVSGFVFLRLICPAILNPRMFNIISDSPSPIAARTLILVAKSVQNLANLVEFGAKEPYMEGVNPFIKSNKHRMIMFLDELGNVPELPDTTEHSRTDLSRDLAALHEICVAHSDELRTLSNERGAQQHVLKKLLAITELLQQKQNQYTKTNDVR		MHHHHHHGSLVPRSENLYFQGSTAPPTNQWYHGKLDRTIAEERLRQAGKSGSYLIRESDRRPGSFVLSFLSQMNVVNHFRIIAMSGDYYIGGRRFSSLSDLIGYYSHVSSLLKGEKLLYPVAPPEPVEDRRRVRAILPYTKVPDTDEISFLKGDMFIVHNELEDGWMWVTNLRTDEQGLIVEDLVEEVGREEDPHEGKIWFHGKISKQEAYNLLMTVGQVSSFLVRPSDNTPGDYSLYFRTNENIQRFKISPTPNNQFMMGGRYYNSIGDIIDHYRKEQIVEGYYLKEPVPMQDQEQVLNDTVDGKEIYNTIRRKTKDAFYKNIVKKGYLLKKGKGKRWKNLYFILEGSDAQLIYFESEKRATKPKGLIDLSVCSVYVVHDSLFGRPNCFQIVVQHFSEEHYIFYFAGETPEQAEDWMKGLQAFCNLRKSSPGTSNKRLRQVSSLVLHIEEAHKLPVKHFTNPYCNIYLNSVQVAKTHAREGQNPVWSEEFVFDDLPPDINRFEITLSNKTKKSKDPDILFMRCQLSRLQKGHATDEWFLLSSHIPLKGIEPGSLRVRARYSMEKIMPEEEYSEFKELILQKELHVVYALSHVCGQDRTLLASILLRIFLHEKLESLLLCTLNDREISMEDEATTLFRATTLASTLMEQYMKATATQFVHHALKDSILKIMESKQSCELSPSKLEKNEDVNTNLTHLLNILSELVEKIFMASEILPPTLRYIYGCLQKSVQHKWPTNTTMRTRVVSGFVFLRLICPAILNPRMFNIISDSPSPIAARTLILVAKSVQNLANLVEFGAKEPYMEGVNPFIKSNKHRMIMFLDELGNVPELPDTTEHSRTDLSRDLAALHEICVAHSDELRTLSNERGAQQHVLKKLLAITELLQQKQNQYTKTNDVR	

Peptide Bound	N/A	GTKV[pTyr]IDPFT[pTyr]EDPNEA	SD[pTyr]AEPMDAVVKPRNEEENI[pTyr]SVPH	N/A	GTKV[pTyr]IDPFT[pTyr]EDPNEA	SD[pTyr]AEPMDAVVKPRNEEENI[pTyr]SVPH	SPPAL[pTyr]AEPLDSLRIAPCPSQDSL[pTyr]SDPLDST
Extinction coefficient ε (M^{-1} cm^{-1})	45840			101035			
MW (kDa)	31.4	33.6	34.9	103.4	105.6	106.9	107
Loading concentration (mg mL^{-1})	3.8	7.6	3.8	2.4	2.4	2.4	3.7
Injection volume (µL)	250	250	320	300	300	300	175
Flow Rate (ml min^{-1})	0.5			0.6			
Solvent composition	20 mM Tris pH 8 350 mM NaCl 1 mM DTT			20 mM Tris pH 8 150 mM NaCl 1 mM DTT			
**SAXS Data Collection Parameters**							
Instrument	BioCAT (Sector 18, APS)						
Wavelength (Å)	1.033						
Camera length (m)	3.628			3.69		3.653	
Beam size	150 (h) x 25 (v) focused at the detector						
q-measurement range (Å-1)	0.0045 to 0.35			0.003 to 0.35	0.003 to 0.35	0.0029 to 0.42	0.0029 to 0.42
Absolute scaling method	Glassy Carbon, NIST SRM 3600						
Basis for normalization to constant counts	To transmitted intensity by beam-stop counter						
Method for monitoring radiation damage	Automated frame-by-frame comparison of relevant regions using CORMAP (Franke et al., 2015)(77) implemented in BioXTAS RAW						
Sample configuration	SEC-MALS-DLS-RI-SAXS. Size separation used a Superdex 200 Increase 10/300 GL column (Cytiva) and a 1260 Infinity II HPLC (Agilent Technologies). UV data was measured in the Agilent, and MALS-DLS-RI data by DAWN HELEOS-II (17 MALS + 1 DLS channels) and Optilab T-rEX (RI) instruments (Wyatt). SAXS data was measured in a sheathflow cell (Kirby et al., 2016)(75), effective path length 0.542 mm.						
Exposure time (s)	0.5			0.7		0.5	
Exposure period (s)	2			1			
Sample temperature (˚C)	22			20		22	
**Software employed for SAXS data reduction**							
SAXS data reduction	Radial averaging; frame comparison, averaging, and subtraction done using BioXTAS RAW 2.0.3 (Hopkins et al., 2017)(76)						
Basic analysis: Guinier, M.W., P(R)	Guinier fit and M.W. using BioXTAS RAW, P(r) function using GNOM (Svergun, 1992)(78). RAW uses MoW and Vc M.W. methods (Rambo & Tainer, 2013; Piiadov et al., 2018)(79,80)						
ε from sequence	ProtParam Tool - ExPASy						
Electron density				Performed in RAW v2.1.3 according to (Grant 2018)(81)			
Molecular graphics				CCP4mg			

**Structural parameters**							
Guinier Analysis							
I(0) (cm^{-1})	0.00581	0.0163	0.114	0.0257	0.0232	0.0122	0.0150
R_g (Å)	25.12	26.78	26.78	39.47	39.2	40.13	40.49
q-range (Å^{-1})	16 – 153	0 - 143	0 - 143	0 - 87	5 - 87	12 - 228	0 - 216
P(R) Analysis							
R_g (Å)	25.77	27.42	24.03	39.82	39.6	41.15	40.92
D_{max} (Å)	98	105	77	140	137	151	146
q-range (Å^{-1})	0.009070167 - 0.352584764	0.0044631 - 0.35258476	0.004463098 - 0.352584764	0.00297235 - 0.34549988	0.00438775 - 0.34549988	0.00442436 - 0.42367954	0.0029288 - 0.42367954
Porod Volume (Vp) MW (kDa)	34.2	36.1	32.9	118.8	129.0	126.6	126.7
Volume of Correlation (Vc) MW (kDa)	31.1	32.1	31.0	100.29	116.64	108.91	107.49
**DENSS Reconstructions**							
q-range for fitting				0.00297235 - 0.34549988			
Symmetry/anisotropy assumptions				N/A			
Ambiguity measure				2.238			
Chi squared value				0.04752			
Model R_g (Å)				38.53			
Model resolution (Å)				48 ± 11			
**Data and model deposition IDs**	SASDRJ6	SASDRK6	SASDRL6	SASDRE6	SASDRF6	SASDRG6	SASDRH6
**Frames Averaged**	963-1040	978-1079	978-1079	1343-1407	1351-1422	1376-1422	1367-1413
**Buffer Frames Used**	490-629	621-782	621-782	961-1059	586-800	405-558	297-430

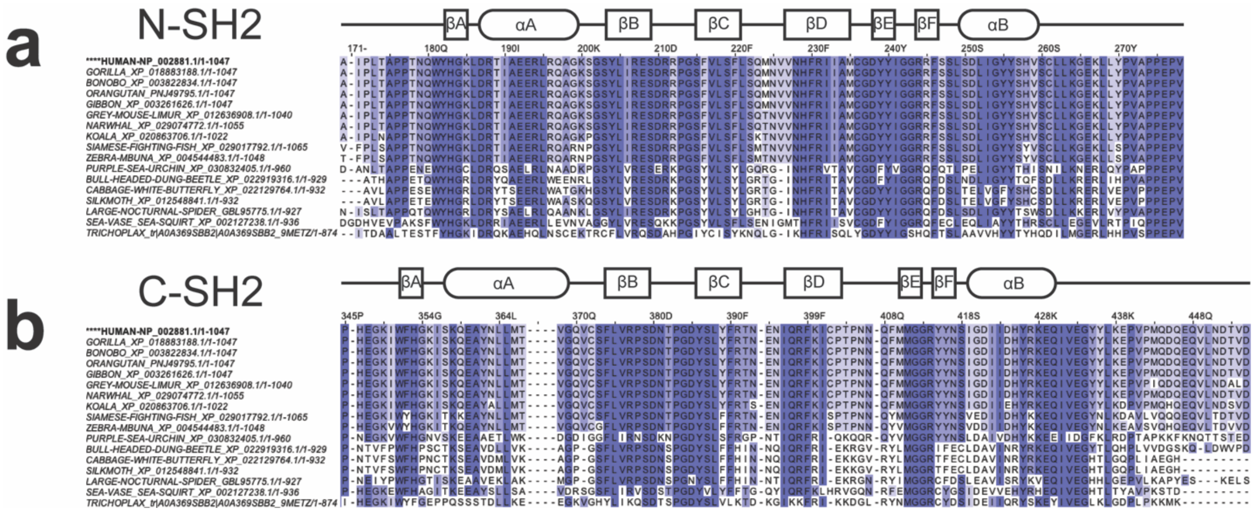

Figure S1. Sequence alignment of RasGAP's N- and C-SH2 domains from different species. a) Represents N-SH2 and **b)** represents C-SH2 domains of human (Uniprot ID P20936); gorilla (Uniprot ID A0A2I2Z1Q1); bonobo (Uniprot ID A0A2RAHG3); orangutan (Uniprot ID H2PG18); gibbon (Uniprot ID G1RT91); lemur (Uniprot ID A0A8B7HI03); narwhal (Uniprot ID A0A8B7HI03); koala (Uniprot ID A0A6P5M668); Siamese fighting fish (Uniprot ID A0A6P7NDR8); zebra (Uniparc ID UPI000329D729); sea urchin (Uniprot ID A0A7M7N7P5) dung beetle (Uniparc ID UPI000C200BA4); butterfly (Uniparc ID UPI000B92D6D2); silk moth (Uniprot ID A098R2GBA6); spider (Uniprot ID A0A4Y2BWC2); sea squirt (Uniparc ID UPI000052352E); and Trichoplax (Uniprot ID A0A369SBB2). Alignment was made using MAFFT (86) and visualized using Jalview (88).

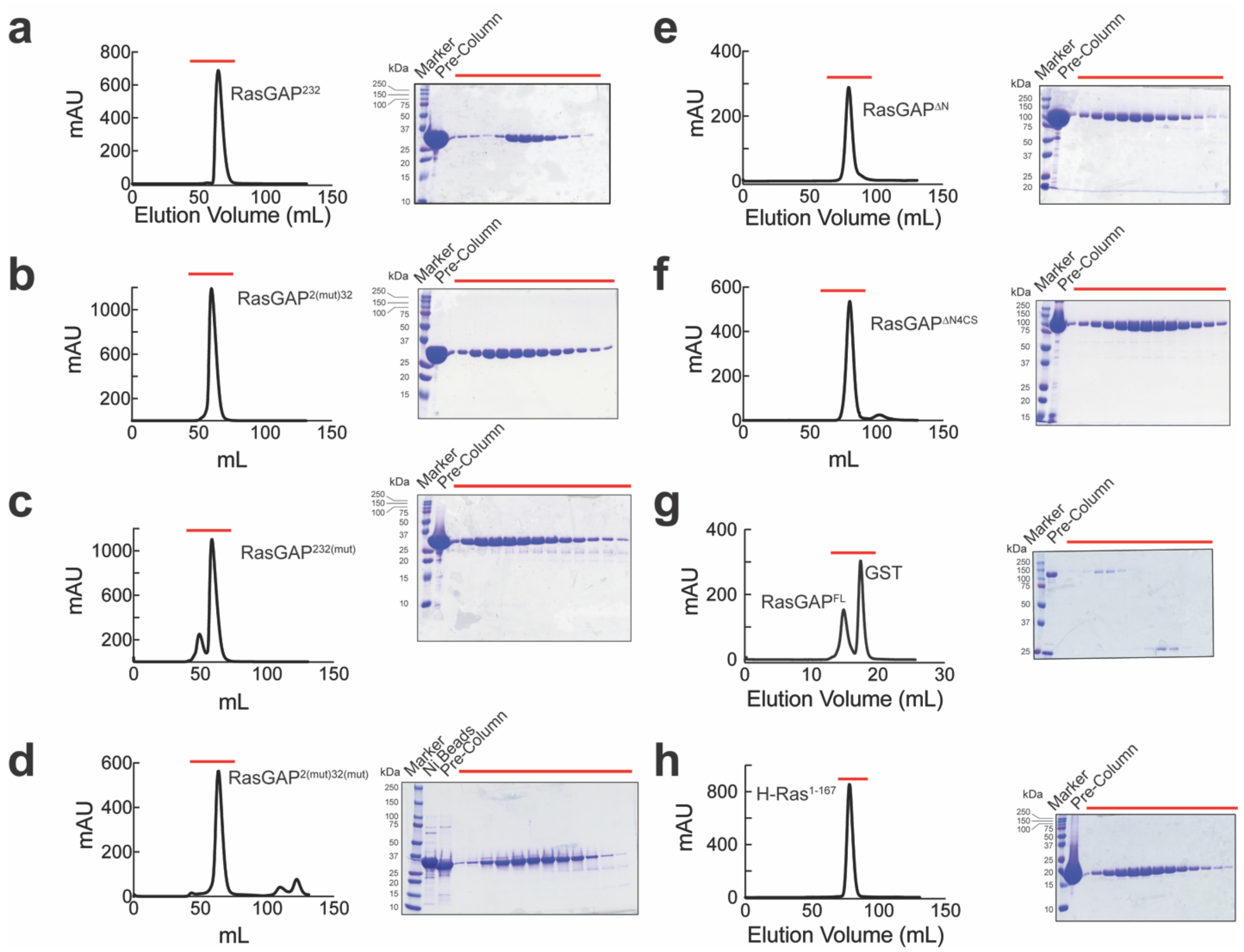

Figure S2. **Purification of all protein constructs used in this study. a)** RasGAP^{232} purified via size exchange chromatography (SEC) using a Hi Load Superdex 75 16/600 prep grade column. Eluted fractions were run on a 15% SDS-PAGE gel (indicated by red bar). **b)** RasGAP$^{2(mut)32}$ purified via size exchange chromatography (SEC) using a Hi Load Superdex 75 16/600 pg column. Eluted fractions were run on a 15% SDS-PAGE gel (indicated by red bar). **c)** RasGAP$^{232(mut)}$ purified via size exchange chromatography (SEC) using a Hi Load Superdex 75 16/600 pg column. Eluted fractions were run on a 15% SDS-PAGE gel (indicated by red bar). **d)** RasGAP$^{2(mut)32(mut)}$ purified via size exchange chromatography (SEC) using a Hi Load Superdex 75 16/600 pg column. Eluted fractions were run on a 15% SDS-PAGE gel (indicated by red bar). **e)** RasGAP$^{\Delta N}$ purified via size exchange chromatography (SEC) using a Hi Load Superdex 200 16/600 pg column. Eluted fractions were run on a 12% SDS-PAGE gel (indicated by red bar). **f)** RasGAP$^{\Delta N4CS}$ contains cysteine to serine mutations to ensure protein homogeneity by inhibiting disulfide-linked multimers via surface-exposed Cys. This protein was purified via size exchange chromatography (SEC) using a Hi Load Superdex 200 16/600 pg column. Eluted fractions were run on a 12% SDS-PAGE gel (indicated by red bar). **g)** Full length RasGAP size exchange chromatography (SEC) purification

performed on a GE Superdex 200 increase 10/300 GL column. Free GST elutes after the RasGAP peak. 12% SDS-PAGE of fractions from peak (indicated in red). **h)** H-Ras SEC purification performed on a GE HiLoad 16/600 Superdex 75 pg column. 18% SDS-PAGE of fractions from H-Ras SEC peak (indicated in red).

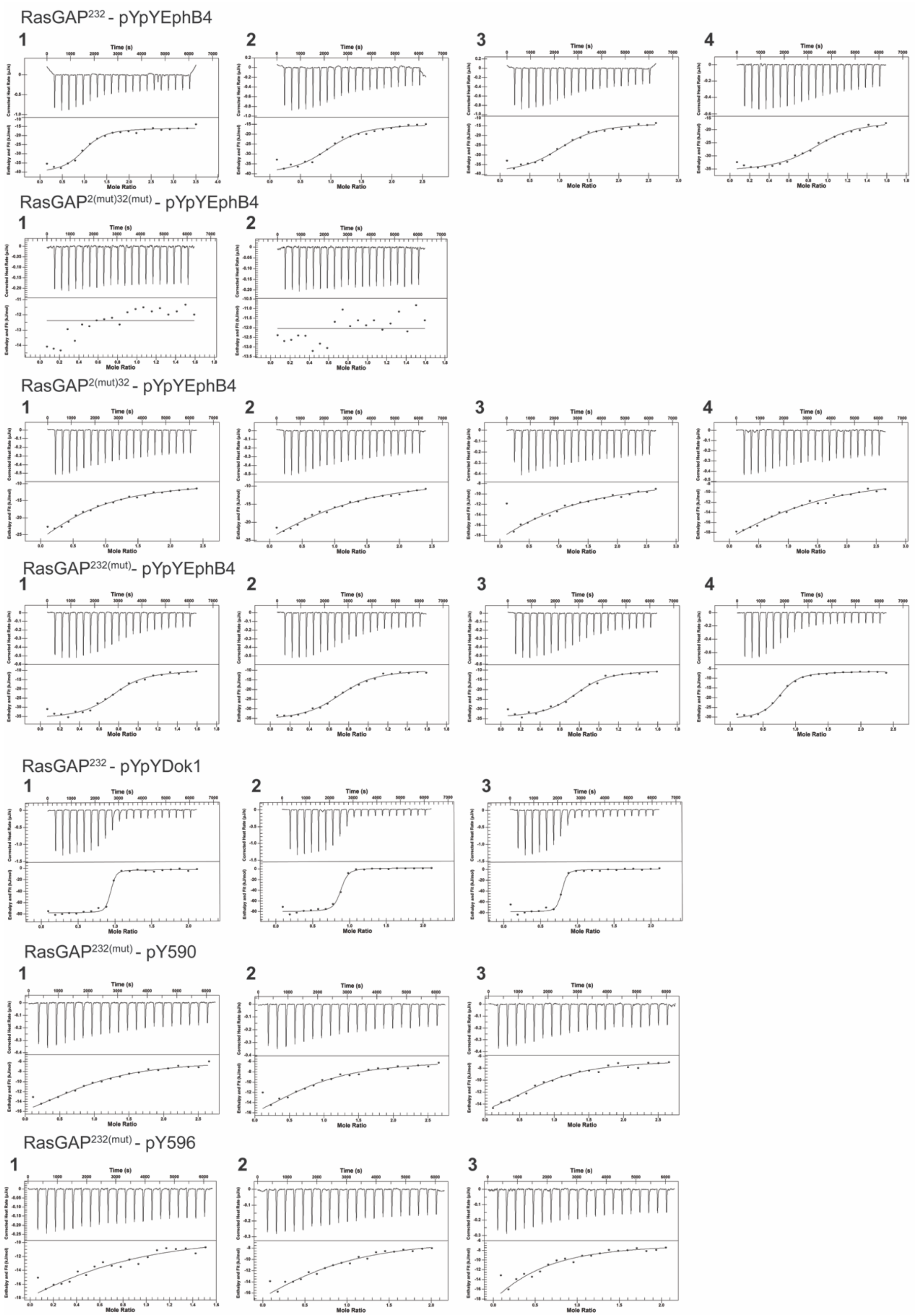

Figure S3. Thermograms and binding isotherms of Isothermal Titration Calorimetry trials used in Table 1. The trial number associated with Supplementary Table 1 is indicated to the top left of each

thermogram. Two to four trials were performed for each binding pair tested. Thermograms shown in Figure 2 are reproduced here to allow side-by-side assessment of each run. These trials are: RasGAP^{232} – pYpYDok1 Trial 3; RasGAP^{232} – pYpYEphB4 Trial 3; RasGAP$^{2(mut)32(mut)}$ – pYpYEphB4 Trial 2; RasGAP$^{232(mut)}$ – pYpYEphB4 Trial 2; RasGAP$^{2(mut)32}$ – pYpYEphB4 Trial 2; RasGAP$^{232(mut)}$ – pY590 Trial 2; RasGAP$^{232(mut)}$ – pY596 Trial 2.

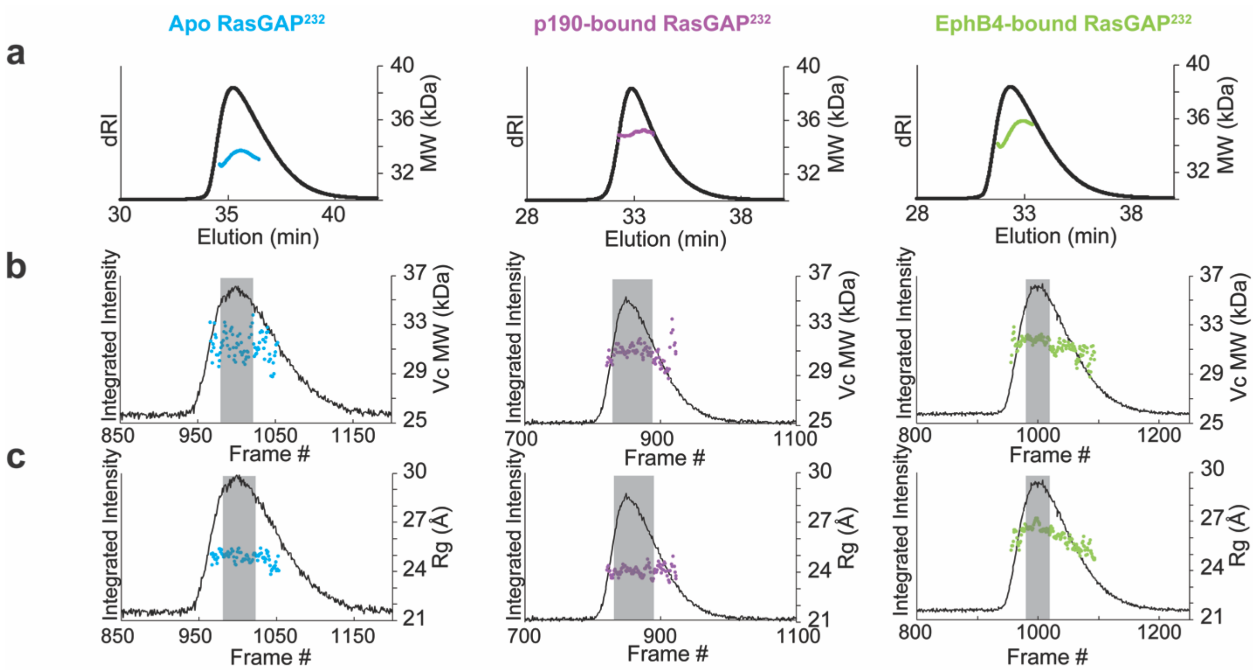

Figure S4. In-line SEC-MALS-SAXS analysis of RasGAP^{232} samples. a) Multi Angle Light Scattering (MALS) analysis of protein peaks. The average molecular weight estimate agrees with the theoretical molecular weight of each sample. **b) and c)** Integrated intensity of each scattering frame collected over the length of protein elution. The **(b)** volume of correlation (V_c) and **(c)** R_g are plotted for each frame. Dark bands indicate frames averaged to make scattering profile in **Figure 3a**.

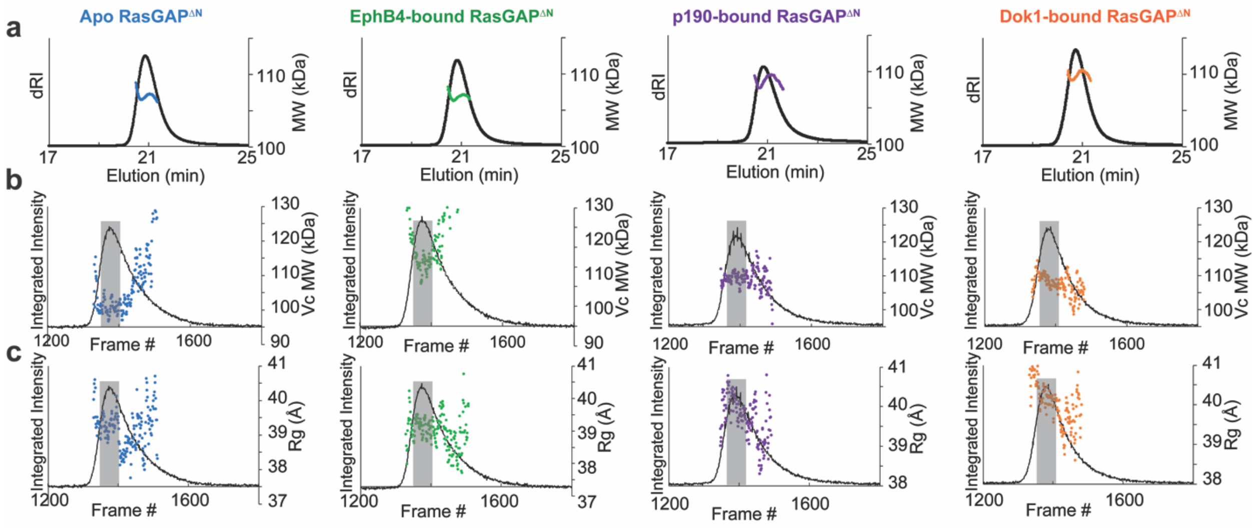

Figure S5. In-line SEC-MALS-SAXS analysis of all RasGAP$^{\Delta N}$ samples. a) Multi Angle Light Scattering (MALS) analysis of protein peak. The average molecular weight estimates match the theoretical values for each protein monomer. **b) and c)** Integrated intensity of each scattering frame collected over the length of protein elution. The **(b)** volume of correlation (V_c) and **(c)** R_g are plotted for each frame. Dark bands indicate frames averaged for scattering profiles in **Figures 4a and 5a**.
